# Who wins? Analysis and Simulation of a Batch Culture Model of Bacterial Competition in the Presence of Plasmids

**DOI:** 10.1007/s11538-026-01708-1

**Published:** 2026-07-22

**Authors:** Ana C. Mendez, Fawaz K. Alalhareth, LeNaiya Kydd, Maryann E. Hohn, Ami Radunskaya, Justyn Jaworski, Hristo V. Kojouharov

**Affiliations:** 1 Department of Mathematics, The University of Texas at Arlington, Arlington, TX 76019 USA; 2 Department of Mathematics, College of Arts and Sciences, Najran University, Najran, Saudi Arabia; 3 Science and Engineering Research Center, Najran University, Najran, Saudi Arabia; 4 Department of Bioengineering, The University of Texas at Arlington, Arlington, TX 76019 USA; 5 Institute for Defense Analyses, Center for Computing Sciences, Bowie, MD USA; 6 Department of Mathematics, Pomona College, Claremont, CA 91711 USA

**Keywords:** Bacterial growth, Plasmids, *Escherichia coli*, Optical density, Batch culture

## Abstract

Bacteria are essential in research and commercial processes for producing DNA, proteins, and converting raw materials into high-value molecules, often using batch culture systems. These systems provide controlled conditions for bacterial growth, which is influenced by factors like temperature, oxygen, and nutrients. This study introduces a mathematical model of plasmid dynamics, including loss, uptake, and transfer by conjugation, within batch cultures. The model helps optimize *E. coli* cultures for product formation and predict plasmid-carrying bacteria levels, offering insights into plasmid dynamics in “one-pot” systems. Our findings show that plasmid retention is influenced by selection pressures which can be an important consideration in probiotic dosing regimens. The model aligns with experimental data and highlights the importance of understanding plasmid dynamics for controlling bacterial growth processes, with implications for research, commercial applications, and gut microbiome stability. Future work will explore temporal changes in plasmid dynamics, requiring advanced instrumentation for precise bacterial population quantification.

## Introduction

Bacteria are widely used within research and commercial processes for the production of DNA, proteins, or the conversion of raw materials into high value molecules. To obtain such products, bacteria are often grown in batch culture which is a system that provides a “one-pot” approach to supplying controlled initial conditions for growth and constant environmental conditions (Planson et al. 2020). Understanding and controlling bacterial growth within the batch culture system is thus critical to obtaining high yields of a desired bacterial product. Bacterial growth can be influenced by a number of different factors such as temperature, accessible oxygen, spatial confinements, and nutrient availability (Donachie et al. 1976; Grossman et al. 1982; Allen and Waclaw 2018). In batch culture systems, a small number of cells are inoculated in a container filled with liquid nutrient medium, which is not replenished, and that is then allowed to incubate under continuous shaking conditions at a constant temperature. During the batch culture period, the bacterial cells divide by means of binary fission, which will occur rapidly under optimal conditions (Allen and Waclaw 2018), and the concentration of viable cells within the system follows a definitive course of growth with extended incubation time (Baranyi et al. 1993).The growth dynamics consists of the following four different phases that reflect the physiological state of the bacteria in the culture at that particular time: 1. Lag phase, 2. Exponential phase, 3. Stationary phase, 4. Death phase (Allen and Waclaw 2018; Baranyi et al. 1993; Rolfe et al. 2012).

Analysis tools such as spectrophotometers are used to quantify the concentration of bacteria along these growth curves in order to identify which phase the bacteria are experiencing (Zwietering et al. 1990). Determining the growth phase is important, for example, in correctly timing the mid-log phase during which biochemical inducers that initiate protein production are added when using expression systems that contain an inducible promoter. In this scenario, the bacteria are allowed to grow to a sufficient concentration, and while undergoing exponential growth, the biochemical inducer is provided that causes the bacteria to dedicate themselves to protein production. Any metabolic burden upon the bacteria, such as high levels of protein production, will slow their growth; thus, it is necessary the batch culture first achieve a sufficient density of cells when inducing protein production while not yet reaching the stationary phase wherein resources are limited. Optimization of the density of viable cells in batch cultures can thereby dictate the level of efficiency of the batch culture in terms of product yield.

For engineering of bacterial systems to create a desired product, researchers often transform bacteria using plasmids which serve as carriers of genetic information. Plasmids are extra-chromosomal DNA molecules that are dynamically taken up by cells, transferred to other cells by conjugation, or lost from cells. Each of these processes can result in changes in the cell’s behavior by gaining or losing genes with specific functions. Maintenance of plasmid replication within a cell often incurs a metabolic burden that is attributed to the level of its replication and production of any proteins encoded by genes carried on the plasmid. This metabolic burden can become greater when a high number of copies of the plasmid are produced at each binary fission cycle resulting in slower rates of bacterial replication. Bacteria possessing higher metabolic burden will inherently replicate more slowly and thus be surpassed by faster replicating bacteria having less metabolic burden if holding other factors equal. Maintenance of plasmid replication can nonetheless have its benefits, as expression of plasmid-derived genes may provide the host bacteria with important functions contributing to a survival advantage such as antibiotic resistance. Understanding plasmid dynamics within bacterial systems is important to knowing the level of heterogeneity of cells within a culture which can provide information about the behavior of the culture.

Previous mathematical models have been developed to describe interactions between plasmid-carrying bacteria. A theoretical understanding of how plasmids and bacteria interact from a modeling perspective can be found in Dewan and Uecker (2023). Our model is similar to those presented in Webb et al. (2005) and D’Agata et al. (2008), which describe bacteria interacting with immune cells. In D’Agata et al. (2008), a system of equations describing the transfer of plasmid between bacteria is studied in order to understand the impact of acquired antibiotic resistance on the evolution of competing populations. The effect of the plasmid reproduction rate is the focus of a similar model in Ibargüen-Mondragón et al. (2021), where a third equation is added to the system to explicitly model the plasmid itself.

In this study, we develop a new mathematical model of the dynamics of two cell populations within a batch culture that includes plasmid loss and uptake for both populations. The model also includes transfer by conjugation to provide a representation of the co-existence of plasmid-carrying bacteria and plasmid-free bacteria. In contrast to previous models such as D’Agata et al. (2008), we assume that plasmid-carrying and plasmid-free bacteria have different resource demands.

We expect that our new model will be a useful tool to guide appropriate initial conditions to optimize co-cultures for microbial bioproduction. In addition, this model offers a means for predicting the extent of plasmid-carrying bacteria within cultures. Developing a better understanding of the way in which bacteria retain, lose, or transfer plasmid and its impact on the population of bacterial communities holds importance to refining our ability to predict and control bacterial growth processes that are critical not only to research and commercial applications but also to the stability and health of our gut microbiome.

## Biological Background and Modeling Assumptions

**Fig. 1:**
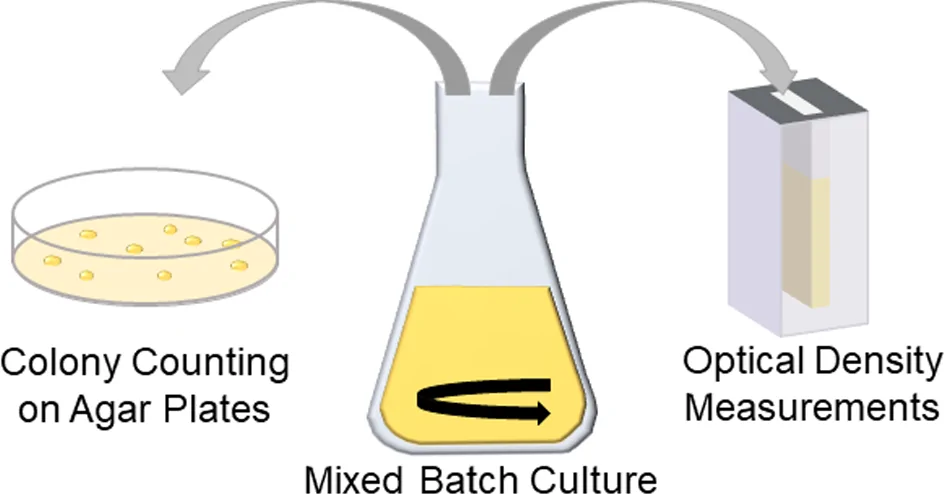
Schematic illustration of the use of culture samples for plate-based colony counting assays or optical density measurements to determine concentration of bacteria in mixed batch cultures over time (color figure online)

**Fig. 2:**
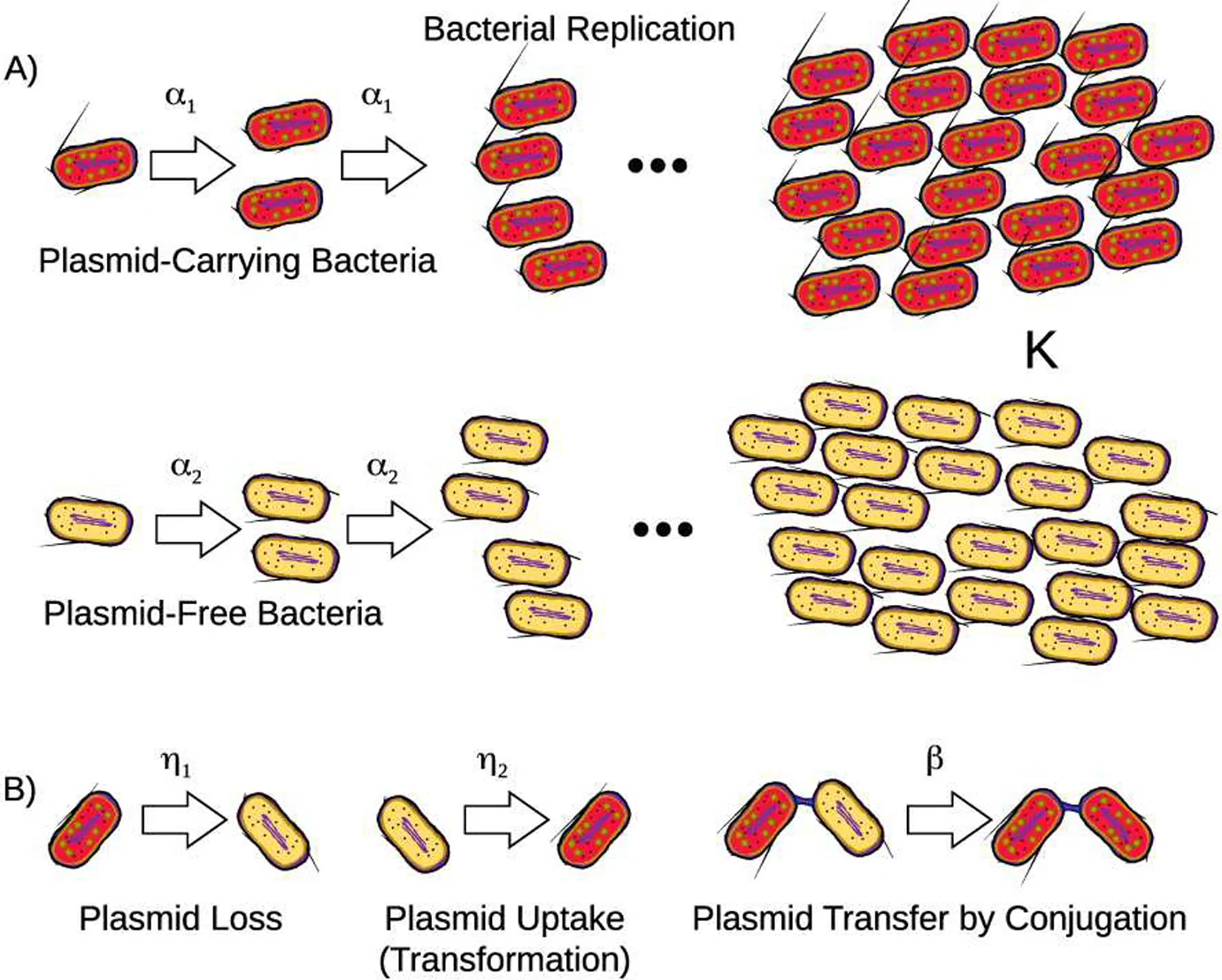
Schematic illustration of A) the processes by which binary fission in bacterial replication provides exponential increase in plasmid-carrying and plasmid-free bacteria and B) the processes by which plasmids can be lost or gained (i.e., transformation and conjugation) by bacteria (color figure online)

To begin to understand the population dynamics occurring between plasmid-carrying bacteria and plasmid-free bacteria, we conducted individual batch culture experiments to observe bacterial growth (Figure 1). As a model organism for exploring microbiology and one of the most common engineered organisms in biotechnology, we utilized the bacteria *E. coli* and quantified its growth in batch cultures for those which possessed a plasmid (plasmid-carrying) as well as those which did not possess a plasmid (plasmid-free). From here, we garnered an initial understanding of plasmid maintenance, specifically accounting for metabolic burden of plasmid replication on the host growth rate. In batch cultures, an important differentiation between the bacteria is whether a given bacteria still carries the plasmid or if it is plasmid-free which gives an indication of the heterogeneity of the culture. If ignoring environmental selection, the extent of plasmid persistence within a bacterial population during batch culture will depend on rates of replication of the plasmid-free bacteria, plasmid-carrying bacteria, rate of plasmid loss, rate of plasmid uptake (transformation of a previously plasmid-free bacteria with a freely available plasmid), and rate of plasmid transfer by conjugation from a plasmid-carrying bacteria to a previously plasmid-free bacteria (Figure 2).

Plasmid loss at cell division, in which all copies of the plasmid separate to only one daughter cell after binary fission, can occur and risks elimination of the plasmid over time especially if the plasmid does not encode genes with benefits to host survival or incurs high metabolic burden as the plasmid-free bacteria’s faster replication rate can outcompete plasmid-carrying cells. In the absence of selection pressure, accumulation of mutations in a plasmids origin of replication can decrease efficiency of replication and segregation to both daughter cells thereby causing an increase in the rate of plasmid loss. This rate of plasmid loss can be controlled by utilizing selection pressure such as antibiotics or nutrient deficient media if a survival advantage to maintain the plasmid is apparent through the plasmid carrying genes for antibiotic resistance or enhancing metabolic functions to negate the nutrient deficiency, respectively. It is important to consider that both the mechanism by which an antibiotic acts upon a cell as well as the mechanism of resistance provided by antibiotic resistance genes can differ substantially depending on the class of antibiotic and the manner in which it slows growth or directly kills the bacteria. Because the variability in these interactions can have high heterogeneity in their impact on either individual growth rates or cell death depending on the respective mechanisms, here we take an abstraction from this complexity to focus specifically on plasmid transfer, plasmid retention, and how these are impacted by the presence of antibiotic as a selection pressure. Literal plasmid loss by release of plasmid from bacteria can occur passively by lysis which represents cell death resulting in freeing of plasmid into the environment. Non-lethal plasmid release can also occur actively in a bacterial dependent manner as mediated by secretion systems (Figure 2-B).

In contrast to plasmid loss, bacteria may gain a plasmid by several processes (Figure 2-B). One such process is the uptake of free plasmid from the environment by natural transformation which represents a chance event of plasmid movement into the bacteria that depends not on the plasmid (aside from its concentration in the environment) but on the intrinsic competence of a given bacterial species. This competence is contingent upon the existence of membrane-anchored dsDNA binding proteins that enable passage of DNA into the bacteria and is found to be dependent on cell type and, for lab cultures, also depends on the phase of cell growth (Johnsborg et al. 2007). The second mechanisms accounted for in this model by which a plasmid-free bacteria may receive a plasmid is through the process of conjugation, wherein a bacteria possessing a transferable plasmid makes direct contact with a recipient bacteria utilizing its pilus to form a mating pair. As the pilus retracts to draw the bacteria closer, a channel is formed which permits transfer of the plasmid as a single strand only in the direction toward the recipient bacteria which did not previously possess a pilus. After conjugation, both bacteria in the mating pair will then possess the plasmid.

In our model, we assume the rate at which a plasmid-carrying bacteria loses its plasmid ($\eta _1$) and the rate at which a plasmid-free bacteria acquires plasmid ($\eta _2$) are constant for the duration of the experiment. Furthermore, there is evidence from the experiments that suggests that there is some metabolic burden for carrying a plasmid which costs the bacteria use of resources, which results in either a slower growth rate for plasmid-carrying bacteria or a lower carrying capacity. In our model we also assume the plasmid-carrying bacteria have both a smaller net growth rate and carrying capacity than the plasmid-free bacteria. This is consistent with our experimental results, discussed in Section 5.

## Model Development

We consider a simple batch culture model of bacterial competition in the presence of plasmids. It represents a competition of a plasmid-carrying bacteria $B_1$ and a plasmid-free bacteria $B_2$ in the presence of a constant plasmid in a homogeneous environment. The model incorporates the modeling assumptions as discussed in Section 2 and accounts for the processes illustrated in Figure 2.

The model consists of the following system of two ordinary differential equations:

3.1 $$\begin{aligned} \begin{array}{rcl} \dfrac{dB_1}{dt}& =& \underbrace{\alpha _1B_1\left( 1-\frac{\gamma B_1+B_2}{K}\right) }_{\begin{array}{c} \text {replication of} \\ \text {plasmid-carrying bacteria} \end{array}} -\underbrace{\eta _1 B_1}_{\begin{array}{c} \text {plasmid} \\ \text {loss} \end{array}} +\underbrace{\eta _2 B_2}_{\begin{array}{c} \text {plasmid} \\ \text {uptake} \end{array}} +\underbrace{\beta B_1B_2}_{\begin{array}{c} \text {plasmid transfer by} \\ \text {conjugation} \end{array}},\\ \\ \dfrac{dB_2}{dt}& =& \underbrace{\alpha _2B_2\left( 1-\frac{\gamma B_1+B_2}{K}\right) }_{\begin{array}{c} \text {replication of} \\ \text {plasmid-free bacteria} \end{array}} +\underbrace{\eta _1 B_1}_{\begin{array}{c} \text {plasmid} \\ \text {loss} \end{array}} -\underbrace{\eta _2 B_2}_{\begin{array}{c} \text {plasmid} \\ \text {uptake} \end{array}} -\underbrace{\beta B_1B_2}_{\begin{array}{c} \text {plasmid received} \\ \text {by conjugations} \end{array}}, \end{array} \end{aligned}$$

where $B_1$ and $B_2$ represent the plasmid-carrying bacteria that produces the plasmid and the plasmid-free bacteria, respectively. We assume that bacteria follow a logistical growth with carrying capacity *K* (Ibargüen-Mondragón et al. 2019) and relative capacity γ of $B_1$. Let $\alpha _1$ and $\alpha _2$ be the net growth rate constants of bacteria $B_1$ and $B_2,$ respectively. We also assume that the plasmid-carrying bacteria, $B_1$, grows at a slower rate than the plasmid-free bacteria, $B_2$, and therefore, $\alpha _1<\alpha _2$, with γ>1. Moreover, we assume plasmids are constant and homogeneous across the environment. Bacteria $B_2$ takes in the plasmid and becomes $B_1$ at a rate $\eta _2$, whereas $B_1$ eliminates its plasmid and becomes $B_2$ at a rate $\eta _1$. In addition, there is transfer of plasmid from $B_1$ to $B_2$ by conjugation at a rate β.

The model parameters with their descriptions and units are listed in Table 1.

**Table 1:** Model parameters with their descriptions and units

Variable/Parameter	Description	Units
$B_1$	Plasmid-carrying bacteria	cells/μL
$B_2$	Plasmid-free bacteria	cells/μL
$\alpha _1$	Net growth rate of plasmid-carrying bacteria	1/hour
$\alpha _2$	Net growth rate of plasmid-free bacteria	1/hour
*K*	Carrying capacity of the environment	cells/μL
γ	Relative capacity coefficient	−
$\eta _1$	Rate of conversion from plasmid-carrying to plasmid-free bacteria	1/hour
$\eta _2$	Rate of conversion from plasmid-free to plasmid-carrying bacteria	1/ hour
β	Rate of bacterial conjugation	μL/cells/hour

## Model Analysis

In this section, we present the stability and sensitivity analysis of System (3.1) for the different cases based on the rates of bacterial conversion ($\eta _1$ and $\eta _2$) and bacterial conjugation (β).

### Summary of Stability Analysis

**Fig. 3:**
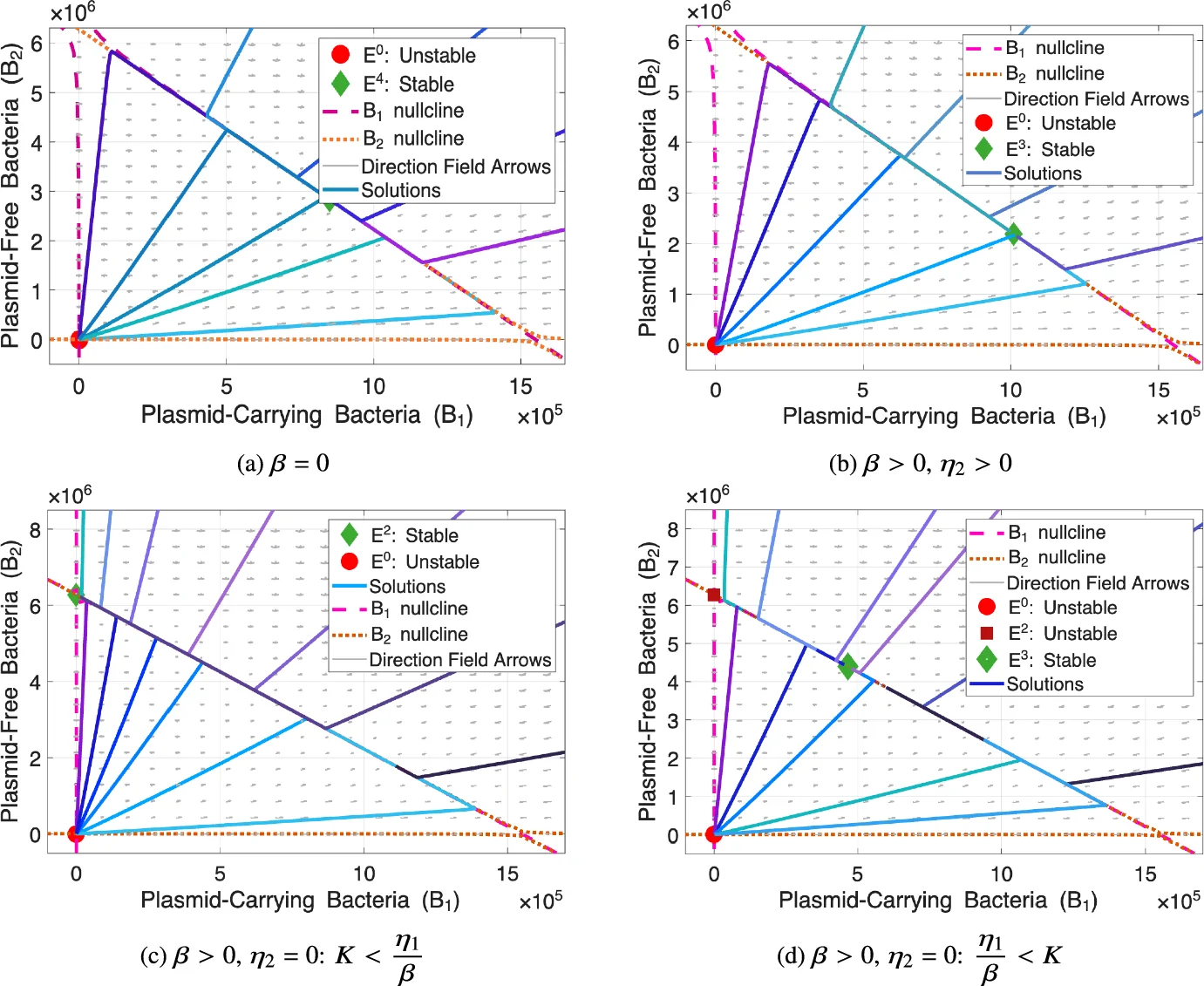
Phase portraits of System (3.1) in each of the different cases of bacterial switching. Parameter values correspond to the four cases described in Tables 2 and 3. In all four panels, we see fast transient behavior towards the slower stable manifold of the stable equilibrium. In panel (a), with β=0, almost all orbits converge towards the co-existing stable equilibrium, although orbits may first show quick growth in either the $B_1$ or $B_2$ populations. In panel (b), with β>0, we see similar behavior, but the stable equilibrium has moved down and to the right, indicating more plasmid-carrying bacteria but fewer plasmid-free bacteria. In panel (c), β>0 but $\eta _2 = 0$ with a carrying capacity smaller than the threshold $\frac{\eta _1}{\beta }$. In this case, the co-existing equilibrium has disappeared, and almost all orbits converge to the stable equilibrium where there are no plasmid-carrying bacteria ($B_1 = 0$). In panel (d), we consider the case when the rate of plasmid loss, $\eta _1$, is smaller relative to the carrying capacity. In this case, we see that the co-existing equilibrium is now stable, and the plasmid-free equilibrium has become unstable (color figure online)

**Fig. 4:**
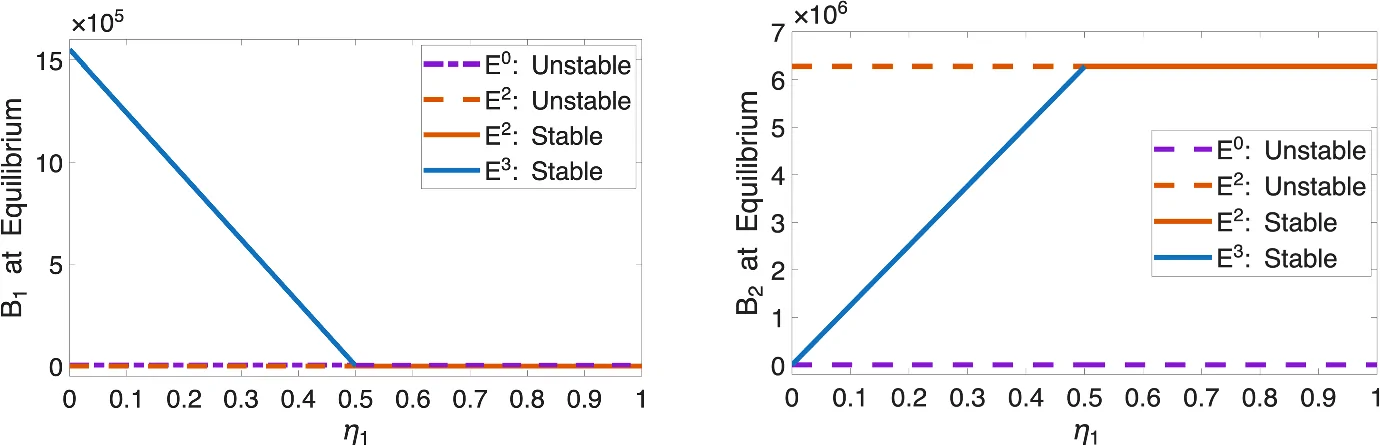
Bifurcation diagrams of system (3.1) when β>0 and ${\eta _2}=0$ for the steady-state plasmid-carrying bacteria $B_1$ (left) and plasmid-free bacteria $B_2$ (right) as the parameter ${\eta _1}$ is varied over the interval $[0, 2\eta _1^{crit}]$, where $\eta _1^{crit} = K \beta$. In the diagrams, solid lines represent stable states while dashed lines represent unstable states. The equilibrium $E^0$ always exists and is unstable, while the equilibrium $E^2$ also always exists but is unstable when $\eta _1 < \eta _1^{crit}$ and locally asymptotically stable when $\eta _1 > \eta _1^{crit}$. The co-existence equilibrium $E^3$ exists only when $\eta _1 < \eta _1^{crit}$ and is locally asymptotically stable when it exists (Table 3) (color figure online)

We present a summary of the stability analysis results for System (3.1) for different cases of bacterial switching, including with (β>0) and without (β=0) transfer of plasmid, respectively. Detailed existence and stability analysis of the equilibria is presented in Appendix A. Phase portraits of System (3.1) in each of the different cases of bacterial switching are displayed in Figure 3, while the bifurcation diagrams are displayed in Figure 4.

#### Case 1: Bacterial switching with transfer of plasmid (β>0)

By setting the right-hand sides of System (3.1) equal to zero, the following three possible biologically-meaningful equilibria are found:

4.1 $$\begin{aligned} E^0 = \left( 0,0\right) \qquad E^1 = \left( \frac{K}{\gamma },0\right) \qquad E^3 =\left( B_1^1,B_2^1\right) , \end{aligned}$$

where

$$\begin{aligned} \displaystyle {B_1^1=\frac{K\beta -(\eta _1+\gamma \eta _2)+\sqrt{4K\gamma \eta _2\beta +(\eta _1+\gamma \eta _2-K\beta )^2}}{2\beta \gamma }}, \\ \displaystyle {B_2^1=\frac{K\beta +(\eta _1+\gamma \eta _2)-\sqrt{4K\gamma \eta _2\beta +(\eta _1+\gamma \eta _2-K\beta )^2}}{2\beta }}. \end{aligned}$$

The existence and stability properties of the above equilibria are different depending on the plasmid-free-to-plasmid-carrying bacteria conversion rate $\eta _2$. Next, we state the results in the corresponding two sub-cases:

- *Bacterial switching with transfer of plasmid and direct acquisition of plasmid *(β>0, $\eta _2 > 0$): When the plasmid-free-to-plasmid-carrying bacteria conversion rate $\eta _2 > 0$, we have only two biologically meaningful equilibria, $E^0$ and $E^3$. Detailed information about $E^0$ in subsection Appendix A.1. Detailed information about $E^1$ in subsection Appendix A.2. Detailed information about $E^3$ in subsubsection Appendix A.4.1. Both equilibria always exist with the trivial equilibrium $E^0$ being unstable while the equilibrium $E^3$ being stable. Note that the equilibrium $E^3$ represents species co-existence only when the plasmid-carrying-to-plasmid-free bacteria conversion rate $\eta _1 > 0$. Otherwise, when $\eta _1 = 0$, our equilibrium $E^3 = E^1 = \left( \frac{K}{\gamma },0\right)$. Table 2 summarizes the existence and stability results, and Figure 3(b) depicts the phase portrait of the corresponding system.
- *Bacterial switching with transfer of plasmid but no direct acquisition of plasmid* (β>0, $\eta _2 = 0$): When the plasmid-free-to-plasmid-carrying bacteria conversion rate $\eta _2 = 0$, all three of the equilibria (4.1) are biologically meaningful and their expressions reduce to the following:
  $$ E^0 = \left( 0,0\right) \qquad E^2 = \left( 0,K\right) \qquad E^3 = \left( \frac{\beta K-\eta _1}{\beta \gamma },\frac{\eta _1}{\beta }\right) . $$
  Their existence and stability properties depend on the relation between the parameters *K*, ${\eta _1}$, and ${\beta }$. In particular, the ratio of the rate of plasmid-carrying-to-plasmid-free bacteria to the rate of transfer of plasmid, $\displaystyle \frac{\eta _1}{\beta }$, in relation to the carrying capacity of the environment, *K*, is the determining factor in the number of equilibria and their corresponding stability. Also, note that the equilibrium $E^3$ represents species co-existence only when the plasmid-carrying-to-plasmid-free bacteria conversion rate $\eta _1 > 0$. Table 3 summarizes the existence and stability results, and Figure 3(c-d) depicts the phase portrait of the corresponding system. Detailed information about $E^0$, $E^2$ and $E^3$ can be found in subsection Appendix A.1, subsection Appendix A.3, and subsubsection Appendix A.4.1, respectively. Next, we visualize the effects of perturbing the value of the rate of plasmid loss ${\eta _1}$ on the number and stability of the steady states of the biological system. Modifications to the rate of plasmid loss $\eta _1$ have direct biological relevance given its ability to be controlled by the degree to which selection pressure is applied to maintain plasmids within a bacterial host. Specifically, the use of antibiotics or nutrient-limited conditions can decrease the rate of plasmid loss if there are genes present on the plasmid that confer the bacteria with antibiotic resistance or with alternative metabolic processing capabilities for nutrient utilization/uptake. Here, we see that when $\eta _1$ is above a critical point ($\displaystyle {\eta _1} > K {\beta }$) the rate of plasmid loss leads to an equilibrium state of the bacteria eventually hosting no plasmid, for example, when there is insufficient selection pressure. Conversely, when $\eta _1$ is below this point ($\displaystyle {\eta _1}< K {\beta }$), the stability of the plasmid within the host bacteria, such as described above for the case of applied selection pressure, leads to an equilibrium of all bacteria being plasmid carriers which would be a desirable case for batch cultures. Figure 4 displays the bifurcation of each equilibrium point, $E^0$, $E^2$, and $E^3$, with respect to the parameter ${\eta _1}$, which is varied in the biologically meaningful interval $[0, 2\eta _1^{crit}]$, where $\eta _1^{crit} = K \beta$, while fixing all other model parameters).

**Table 2:** Existence and local stability results for the equilibrium points of System (3.1) for the sub-case of bacterial switching with transfer of plasmid (β>0) and direct acquisition of plasmid ($\eta _2 > 0$)

**Table 3:** Existence and local stability results for the equilibrium points of System (3.1) for the sub-case of bacterial switching with transfer of plasmid (β>0) but no direct acquisition of plasmid ($\eta _2 = 0$)

#### Case 2: Bacterial switching without transfer of plasmid (β=0)

In this case, System (3.1) reduces to the following system of equation:

4.2 $$\begin{aligned} \begin{array}{rcl} \dfrac{dB_1}{dt}& =& {\displaystyle \alpha _1B_1\left( 1-\frac{\gamma B_1+B_2}{K}\right) }-{\eta _1 B_1} +{\eta _2 B_2},\\ \\ \dfrac{dB_2}{dt}& =& {\displaystyle \alpha _2B_2\left( 1-\frac{\gamma B_1+B_2}{K}\right) }+{\eta _1 B_1} -{\eta _2 B_2}. \end{array} \end{aligned}$$

System (4.2) has only two biologically-meaningful equilibria:

$$ E^0 = \left( 0,0\right) \qquad E^4 = \left( \frac{K\eta _2}{\eta _1+\eta _2\gamma },\frac{K\eta _1}{\eta _1+\eta _2\gamma }\right) , $$

which always exist, with the trivial equilibrium $E^0$ unstable and the equilibrium $E^4$ stable. Note that the equilibrium $E^4$ represents species co-existence only when both conversion rates $\eta _1 > 0$ and $\eta _2 > 0$; otherwise, it represents a boundary equilibrium. Table 4 summarizes the existence and stability results, and Figure 3(a) depicts the phase portrait of the corresponding system. More discussion on these two equilibria can be found at subsection Appendix A.1 and subsubsection Appendix A.4.2.

**Table 4:** Existence and local stability results for the equilibrium points of System (3.1) for the case of bacterial switching without transfer of plasmid (β=0)

##### Remark 4.1

The case of all conversion rates $\beta =\eta _1 =\eta _2 = 0$ corresponds to the basic 2-species Lotka-Volterra interspecific competition model, which has been extensively studied in the scientific literature (eg. Murray (2002)) and, therefore, we are not going to discuss it here.

## Parameter Estimation

To fit the model parameters, we used several experiments that measured cell growth in different conditions using optical density.

### The experimental data

**Fig. 5:**
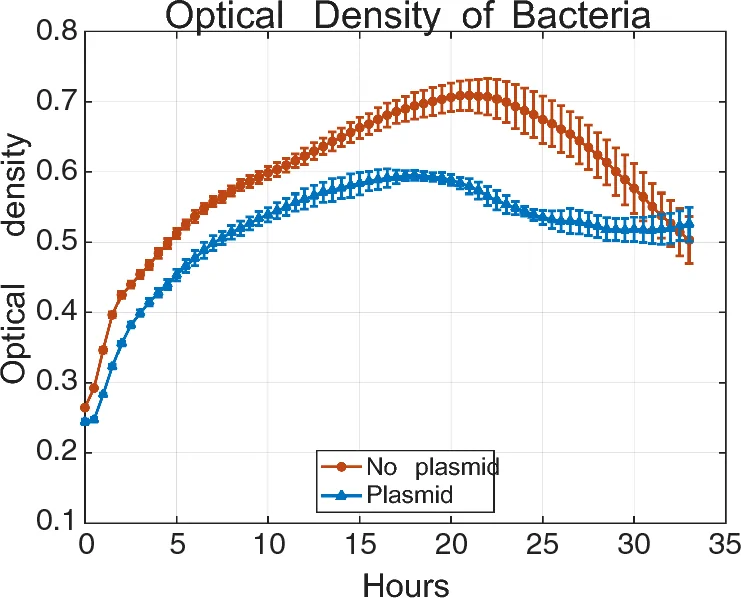
Optical density curves for *in vitro* growth of bacteria with and without plasmid. These curves show how much light is scattered off of a growing culture over a period of thirty hours. The bacteria without plasmid grows more quickly for the first 20 hours (color figure online)

Experimental data consists of optical density measurements over time for bacteria with plasmid and bacteria without plasmid (see Figure 5), along with sparse measurements of colony-forming units (CFU). We used these data to estimate the parameters in Equation (3.1) representing intrinsic bacteria growth, $\alpha _1$ and $\alpha _2$, as well as the carrying capacity, *K*, and the relative consumption rate, γ. We carried out the bacterial growth experiments using *E. coli* grown in Luria-Bertani (LB) broth. Specifically, two culture flasks were provided 20 mL of LB broth and were inoculated with *E. coli* wherein the first flask was provided plasmid-free *E. coli* and the second flask was provided *E. coli* carrying the plasmid P1 which carries a gene conferring antibiotic resistance to ampicillin (Kydd et al. 2022). After overnight culture under shaking conditions of 220 rpm at $37^{\circ }$C, we diluted samples of the overnight culture in fresh LB broth to obtain an optical density near 0.25 (absorbance at 600 nm wavelength of 0.25) for each culture. Using 300 μL of the dilution, we placed the samples in a 96 well flat bottom plate, and we recorded the absorbance at the 600 nm wavelength every 30 minutes with the plate undergoing orbital shaking at room temperature overnight. We recorded the absorbance automatically using an Epoch 2 Bio-TEK microplate reader. For determination of the number of CFUs, separate wells within the same 96 well plate described above were prepared containing the same dilution of bacterial cultures and from which 10 μL of sample were collected periodically. The collected samples underwent sequential one-tenth serial dilution, and 100 μL of those dilutions were placed onto LB agar plates and incubated at $37^{\circ }$C for 24 hours. After 24 hours of growth, the number of colonies present on the plates were counted to approximate the number of cells per microliter within the original liquid cultures within the 96 well plate.

### Bacteria growth kinetics

Recall that our data consists of one bacteria type growing in an environment alone. Thus, with our data, we can estimate parameters that describe one type of bacteria growing in isolation; we have no information about the rates at which plasmid can be transferred between types. To describe the growth of the plasmid-free bacteria, $B_2$, in isolation we let $B_1=0$ and $\eta _2 = 0$ in Equation (3.1). The differential equations then become a single logistic growth equation:

5.1 $$\begin{aligned} \frac{dB_2}{dt} = \alpha _2 B_2 \left( { 1 - \frac{B_2}{K}}\right) \end{aligned}$$

The equation for the plasmid-carrying bacteria is similar, but we have a modified consumption rate:

5.2 $$\begin{aligned} \frac{dB_1}{dt} = \alpha _1 B_1 \left( { 1 - \frac{\gamma B_1}{K}}\right) \end{aligned}$$

As each experiment unfolds, bacteria divide, die, and then the dead cells eventually disintegrate. Since optical density measurements are the result of the total amount of light absorbed, they detect dead cells and cellular debris in addition to living cells. We, therefore, added a differential equation that keeps track of the dead cells, assuming that plasmid-carrying and non-plasmid carrying cells die at fixed rates, denoted by $\delta _1$ and $\delta _2$, respectively. As dying cells undergo lysis, they spill their contents and are no longer in suspension. We represent this process by assuming that both types of dead cells decay, or no longer reflect light, at a rate *d*. This is similar to the approach in Peterson et al. (2025). This gives the following two additional differential equations:

5.3 $$\begin{aligned} \frac{D_1}{dt}= &  \delta _1 B_1 - d D_1 \end{aligned}$$

5.4 $$\begin{aligned} \frac{D_2}{dt}= &  \delta _2 B_2 - d D_2 \end{aligned}$$

We note that bacteria growing *in vitro* eventually consume available nutrients, so that the logistic model as given in Equations (5.2) and (5.1) with a constant carrying capacity, *K*, no longer applies. Therefore, when we fit these models to the optical density measurements shown in Figure 5, we only use data collected in the first 20 hours of the experiment.

### Converting optical density to bacteria cell count

**Fig. 6:**
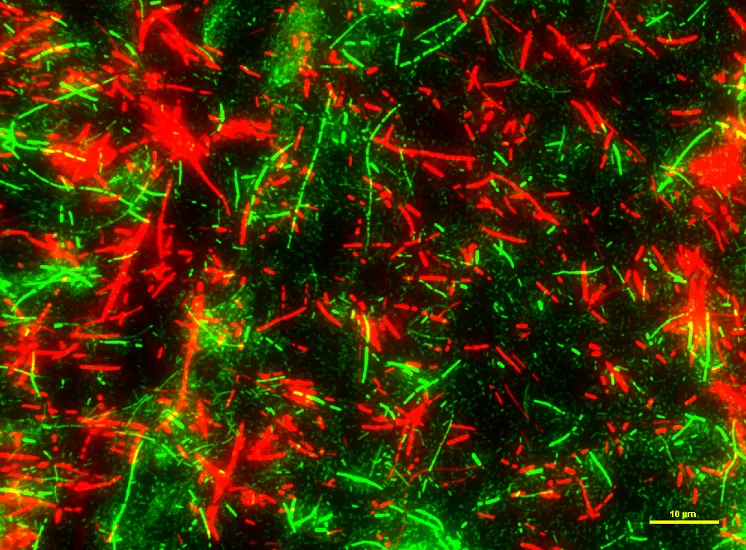
Fluorescence microscopy image of two populations of *E. coli* bearing plasmid expressing either mScarlet or green fluorescent protein showing that cellular chaining phenotypes can occur within cultured cells. Cellular chaining has been previously reported (Vejborg and Klemm 2009), and it may impact optical density measurements due to different orientation and clustering and similarly may significantly influence the number of colony forming units determined within a culture depending on the extent of clustering, which could be affected by factors such as agitation (color figure online)

Optical density (OD) is a common method for estimating cell counts in culture. The OD gives a measurement of how much light is scattered off of a suspension of micro-organisms: the more micro-organisms in the solutions, the more light is scattered, and the higher the OD reading. At moderate densities of the bacteria, the relationship between number of bacteria in the culture and the amount of light scattered is approximately linear; however, the relationship between OD and bacteria number is nonlinear at higher and lower densities (Novak et al. 2009). Furthermore, irregularities in shape, changes in orientation and aggregation of cells affect the quantitative relationship between cell density and the total amount of light scattered (Watson et al. 2004). Figure 6 illustrates how the orientation of cell aggregates at low densities could affect the amount of light that is refracted from a culture. We, therefore, expect that OD measurements at low densities will show a high degree of variability. Since our goal in this project is to understand how two different types of bacteria will grow and interact over a long period of time, this uncertainty in the *in vitro* OD data at very low densities does not affect our results.

The precise relationship between OD and cell count is not generally known, and measurements need to be calibrated for each experimental set-up, taking into account the type of cell, the machine used and the number of wells (Beal et al. 2020). According to Stevenson et al. (2016), for a fixed type of cell and machine, the cell count is a quadratic function of the optical density, while Mira et al find that a fourth degree polynomial gives the best relationship (Mira et al. 2022). In this work, we assume a polynomial relationship of degree less than or equal to four, and fit the coefficients to the data, finding that a polynomial of degree four gives the most likely fit. In other words, if *N* is the total number of cells, we assume that optical density, OD is given by:

5.5 $$\begin{aligned} \textrm{OD} = c_0 + c_1 N + c_2 N^2 + c_3 ^3 + c_4 N^4 \end{aligned}$$

and we fit the parameters $c_0$ through $c_4$ to the data, allowing them to be zero. We note that other relationships, such as quadratic and logarithmic, did not give as good a fit (results not shown).

Since dead cells in suspension are less refractive than living cells, we assume that optical density is a function of the weighted sum:

N=B+wD

where *B* is the density of living cells, *D* is the density of dead cells, and 0<w<1 is a parameter that controls for the loss in refraction of a dead cell compared to a living one.

### Parameter fitting results

We used a Monte Carlo Markov Chain (MCMC) fitting algorithm based on an adaptive Metropolis-Hastings algorithm, implemented in MATLAB©  (code and data available upon request). Considering the number of parameters and the lack of directly observable cell counts, it is not surprising that groups of parameters are correlated and that individual parameters are both structurally and practically unidentifiable. To improve the fitting process, we grouped parameters into identifiable subsets but acknowledge that there are sets in parameter space that would give equally good fits as the ones listed in Tables 5 and 6. We simulated optical density measurements made on simulated bacteria levels and fit them to the experimental OD data shown in Figure 5. We used the MCMC algorithm to find the parameters for both the OD function and the differential equations that maximized the likelihood of the observed OD data. After we obtained rough estimates of the parameters, we ran the MCMC algorithm for one million steps. In each of these later runs, we allowed different subsets of parameters to change in order to explore their correlations.

**Fig. 7:**
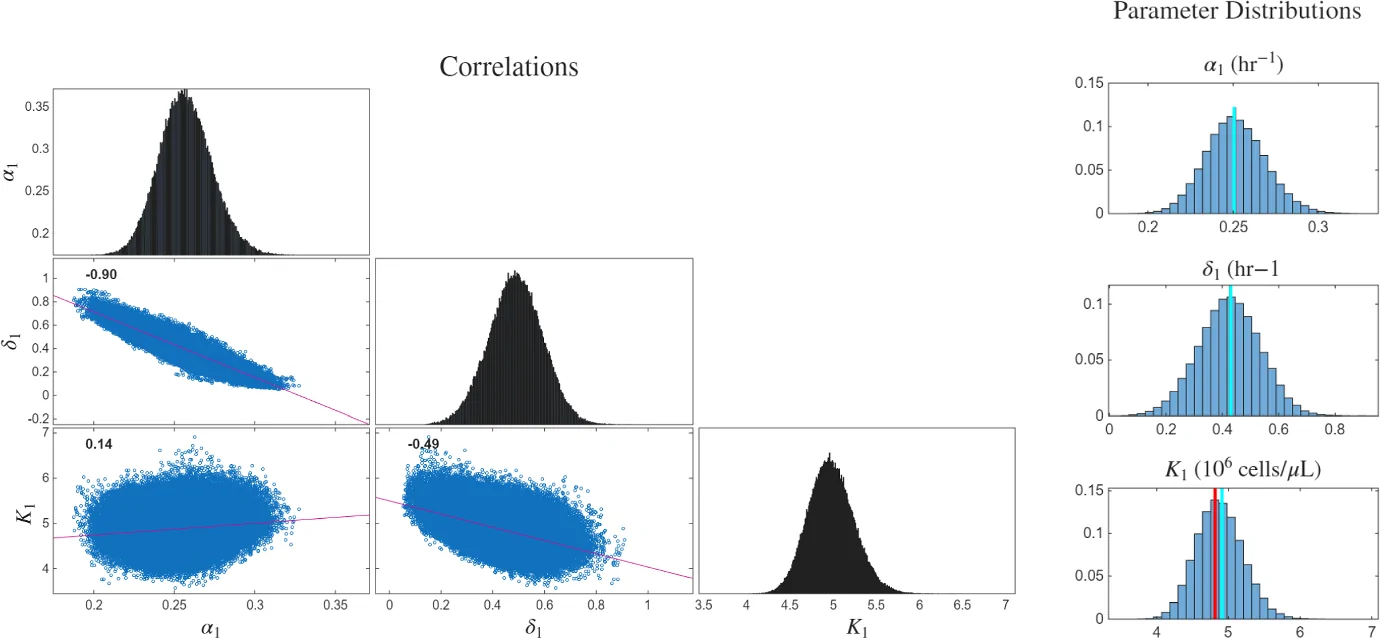
In the left pane above, we show pairwise correlations between the three parameters associated with the plasmid carrying bacteria: $\alpha _1$, $\delta _1$ and $K_1 = K/\gamma$. The growth and death parameters, $\alpha _1$ and $\delta _1$ are strongly negatively correlated, but the carrying capacity, $K_1$ is only weakly correlated with $\delta _1$. The distributions on the right show the uncertainty in parameter estimates, and the shape of these distributions show that the algorithm converged. The cyan bars give the mean of the estimated distribution, which in all three cases is close to the distribution giving the best fit in terms of least-squared distance to the data, which is shown with a red bar. In the case of $\alpha _1$ and $\delta _1$, the cyan and red bars coincide (color figure online)

For example, the left panel of Figure 7 shows correlations between the parameter estimates for the plasmid-carrying bacteria: $\alpha _1$, $\delta _1$ and $K_1$. The growth rate, $\alpha _1$ and the death rate, $\delta _1$, are negatively correlated. This means that, if two sets of parameters have similar OD measurements, the set with the larger value of $\alpha _1$ is likely to have a smaller value of $\delta _1$. This makes sense if one considers that the optical density is a function of a weighted sum of both living and dead cells: N=B+wD. Thus, if the cell growth rate is increased, leading to a larger number of living cells, *B*, the number of dead cells, *D*, must decrease, i.e., the death rate must decrease. On the other hand, the carrying capacity for the plasmid-carrying cells is not correlated with $\alpha _1$ and is only weakly correlated with $\delta _1$.

**Fig. 8:**
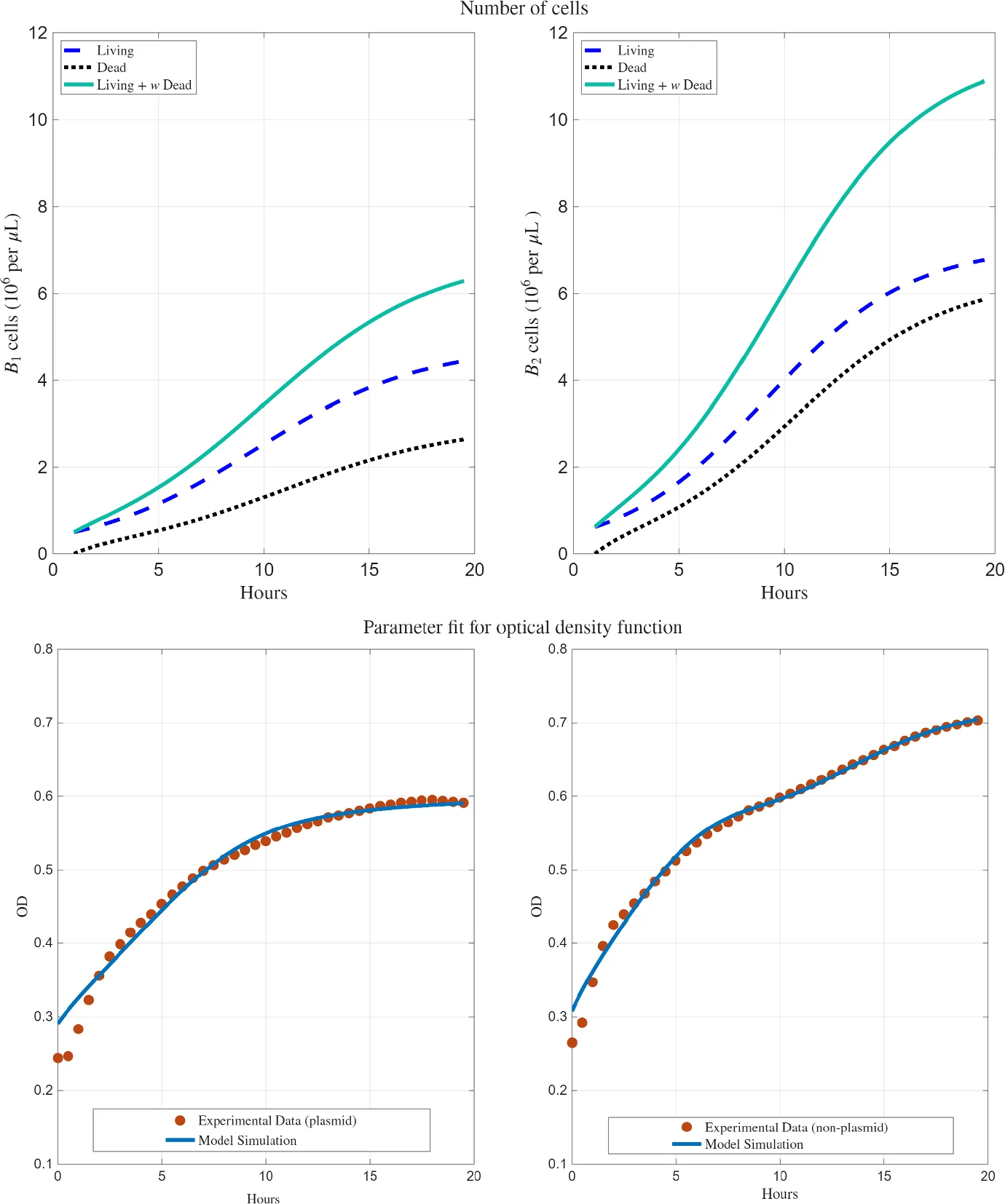
Simulated bacterial growth experiments using parameters fit to laboratory data. Top: *B* = Living (dashed lines) and *D* = Dead cells (dotted lines), along with the weighted sum of both: N=B+wD, showing the different refractory effect of dead cells used in the OD function given in Equation (5.5) (solid lines). Left: plasmid-carrying bacteria ($B_1$); right: non-plasmid carrying bacteria ($B_2$). Bottom: Optical density data in filled red circles; M=model simulation with fitted parameters in solid blue lines (color figure online)

The right panel of Figure 7 shows the estimated distributions of the same parameters: $\alpha _1$, $\delta _1$ and $K_1$. This figure shows that, despite the fact that some parameters are highly correlated, the MCMC procedure has converged, in the sense that the mean of the estimated distributions (the cyan bars in Figure 7) are close to - and sometimes on top of - the values that give the best fit to the data (the red bars in Figure 7). For the simulations shown in Figure 8, we used the parameters corresponding to the best fit which were nearly identical to the estimated mean parameter values.

The simulated bacterial growth curves and the corresponding simulated OD functions are shown in Figure 8. The parameter sets that minimize the squared distance to the OD data from the MCMC runs are given in Tables 5 and 6. We see that both the net growth rate, $\alpha _1$ and the carrying capacity, $K_1 = K/\gamma$, are smaller for the plasmid-carrying bacteria than the corresponding parameters for the plasmid-free bacteria. This is consistent with our expectations based on experimental evidence of the additional metabolic burden due to plasmid maintenance.

**Table 5:** Results of MCMC parameter fitting for the optical density experiments. These parameters describe net growth, death rate, carrying capacity and decay rate. Of particular note is that both the net growth rate and the carrying capacity are smaller for the plasmid-carrying bacteria

Parameter	Plasmid-carrying bacteria ($B_1$)	Value	Plasmid-free bacteria ($B_2$)	Value
Net growth rate	$\alpha _1$	0.25 $\text {hr}^{-1}$	$\alpha _2$	0.30 $\text {hr}^{-1}$
Death rate	$\delta _1$	0.43 $\text {hr}^{-1}$	$\delta _2$	0.62 $\text {hr}^{-1}$
Reflectance loss of dead cells	$w_1$	0.7	$w_2$	0.7
Carrying capacity	$K_1 = K/\gamma$	$4.81 \times 10^6$ cells/μL	$K_2 = K$	$7.11 \times 10^6$ cells/μL
Decay rate	d	0.69 $\text {hr}^{-1}$	d	0.69 $\text {hr}^{-1}$

**Table 6:** Results of the MCMC parameter fitting for the coefficients of the optical density function that describes the mapping from bacteria concentration to OD measurements

OD function coefficient	Value
$c_0$	0.21
$c_1$	0.18
$c_2$	$-3.3 \times 10^{-2}$
$c_3$	$2.7 \times 10^{-3}$
$c_4$	$-7.39 \times 10^{-5}$

## Numerical Simulations

In this section, we perform a series of numerical simulations to explore different biological scenarios with System (3.1). In addition, we present a numerical technique that preserves the key biologically important properties of the model’s solutions, such as positivity and local stability of equilibria, without the need for discretization step size restrictions.

### Second-Order Nonstandard Finite Difference Method

The numerical method introduced below represents a second-order accurate, positivity-preserving, and elementary stable nonstandard (SOPESN) finite difference method, as presented in Alalhareth et al. (2025, 2024). It is based on an extension of the first-order positive and elementary stable nonstandard (PESN) methods from Wood et al. (2015); Wood and Kojouharov (2015), by enhancing their accuracy. In order to apply the SOPESN method, we first rewrite System (3.1) as a productive-destructive system:

6.1 $$\begin{aligned} \begin{array}{rcl} \displaystyle \frac{dB_1}{dt}& =& \underbrace{\alpha _1B_1 +{\eta _2 B_2} +{\beta B_1B_2}}_{P_1(B_1,B_2)} - \underbrace{\Big (\alpha _1B_1\Big (\frac{\gamma B_1+B_2}{K}\Big )+{\eta _1 B_1}\Big )}_{D_1(B_1,B_2)},\\ \\ \displaystyle \frac{dB_2}{dt}& =& \underbrace{\alpha _2B_2 +{\eta _1 B_1}}_{P_2(B_1,B_2)} - \underbrace{\Big (\alpha _2B_2\Big (\frac{\gamma B_1+B_2}{K}\Big )+{\eta _2 B_2} +{\beta B_1B_2}\Big )}_{D_2(B_1,B_2)}, \end{array} \end{aligned}$$

with productive terms:

$$\begin{aligned} P_1(B_1,B_2)&=\alpha _1B_1 +{\eta _2 B_2} +{\beta B_1B_2},\\ P_2(B_1,B_2)&= \alpha _2B_2 +{\eta _1 B_1}, \end{aligned}$$

and destructive terms:

$$\begin{aligned} D_1(B_1,B_2)&=\Big (\alpha _1\Big (\frac{\gamma B_1+B_2}{K}\Big )+{\eta _1}\Big ) B_1,\\ D_2(B_1,B_2)&= \Big (\alpha _2\Big (\frac{\gamma B_1+B_2}{K}\Big )+{\eta _2} +{\beta B_1}\Big )B_2. \end{aligned}$$

Thus, the SOPESN method (Alalhareth et al. 2025) for System (6.1), where $t^i = ih$, with *i* positive integer and numerical step-size h>0, can be written as follows:

6.2 $$\begin{aligned} \begin{array}{rcl} \displaystyle \frac{B_1^{i+1}-B_1^i}{\varphi _1(h,B_1^i,B_2^i)}& =& \alpha _1B_1^i +{\eta _2 B_2^i} +{\beta B_1^iB_2^i}-\Big (\alpha _1\Big (\frac{\gamma B_1^i+B_2^i}{K}\Big )+{\eta _1 }\Big )B_1^{i+1},\\ \\ \displaystyle \frac{B_2^{i+1}-B_2^i}{\varphi _2(h,B_1^i,B_2^i)}& =& \alpha _2B_2^i +{\eta _1 B_1^i} -\Big (\alpha _2\Big (\frac{\gamma B_1^i+B_2^i}{K}\Big )+{\eta _2} +{\beta B_1^i}\Big )B_2^{i+1}, \end{array} \end{aligned}$$

with the denominator function $\varphi _1(h,B_1,B_2)$ and $\varphi _2(h,B_1,B_2)$ are defined as follows

6.3 $$\begin{aligned} \varphi _i(h,B_1,B_2)=\displaystyle h- q_i(B_1,B_2) \frac{h^2}{2}+\mathcal {O}(h^3), \end{aligned}$$

where

$$\begin{aligned} q_1(B_1,B_2)&= -\Big (\frac{\partial P_1(B_1,B_2)}{\partial B_1}-\frac{\partial D_1(B_1,B_2)}{\partial B_1}+ \frac{2D_1(B_1,B_2)}{B_1}\Big )\\ &\quad - \Big (\frac{\partial P_1(B_1,B_2)}{\partial B_2}-\frac{\partial D_1(B_1,B_2)}{\partial B_2}\Big )\Big (\frac{P_2(B_1,B_2)-D_2(B_1,B_2)}{P_1(B_1,B_2)-D_1(B_1,B_2)}\Big ),\\ \\ q_2(B_1,B_2)&= -\Big (\frac{\partial P_2(B_1,B_2)}{\partial B_2}-\frac{\partial D_2(B_1,B_2)}{\partial B_2}+ \frac{2D_2(B_1,B_2)}{B_2}\Big )\\ &-\Big (\frac{\partial P_2(B_1,B_2)}{\partial B_1}-\frac{\partial D_2(B_1,B_2)}{\partial B_1}\Big )\Big (\frac{P_1(B_1,B_2)-D_1(B_1,B_2)}{P_2(B_1,B_2)-D_2(B_1,B_2)}\Big ). \end{aligned}$$

In the numerical simulations, we use the following nonstandard denominator function (Alalhareth et al. 2025):

$$\begin{aligned} \varphi _i(h,B_1,B_2) = \left\{ \begin{array}{lr} \displaystyle \frac{1-e^{-q_i(B_1,B_2)h}}{q_i(B_1,B_2)}, & \text {if } q_i(B_1,B_2)\ne 0\\ \displaystyle h, & \text {if } q_i(B_1,B_2)=0\\ \end{array}\right. , \, \, i=1,2. \end{aligned}$$

### Numerical Explorations of System (3.1)

In this numerical study, we first illustrate the impact of various parameters on the dynamics of the bacterial populations, specifically focusing on the conversion rates between plasmid-carrying ($B_1$) and plasmid-free ($B_2$) bacteria under various conditions and potential treatment strategies. The parameter values used in the numerical simulations for the interaction terms in System (3.1) include γ=4.045, $\eta _1=0.5$, $\eta _2=0.15$, and $\beta =7.974 \times 10^{-8}$.

**Fig. 9:**
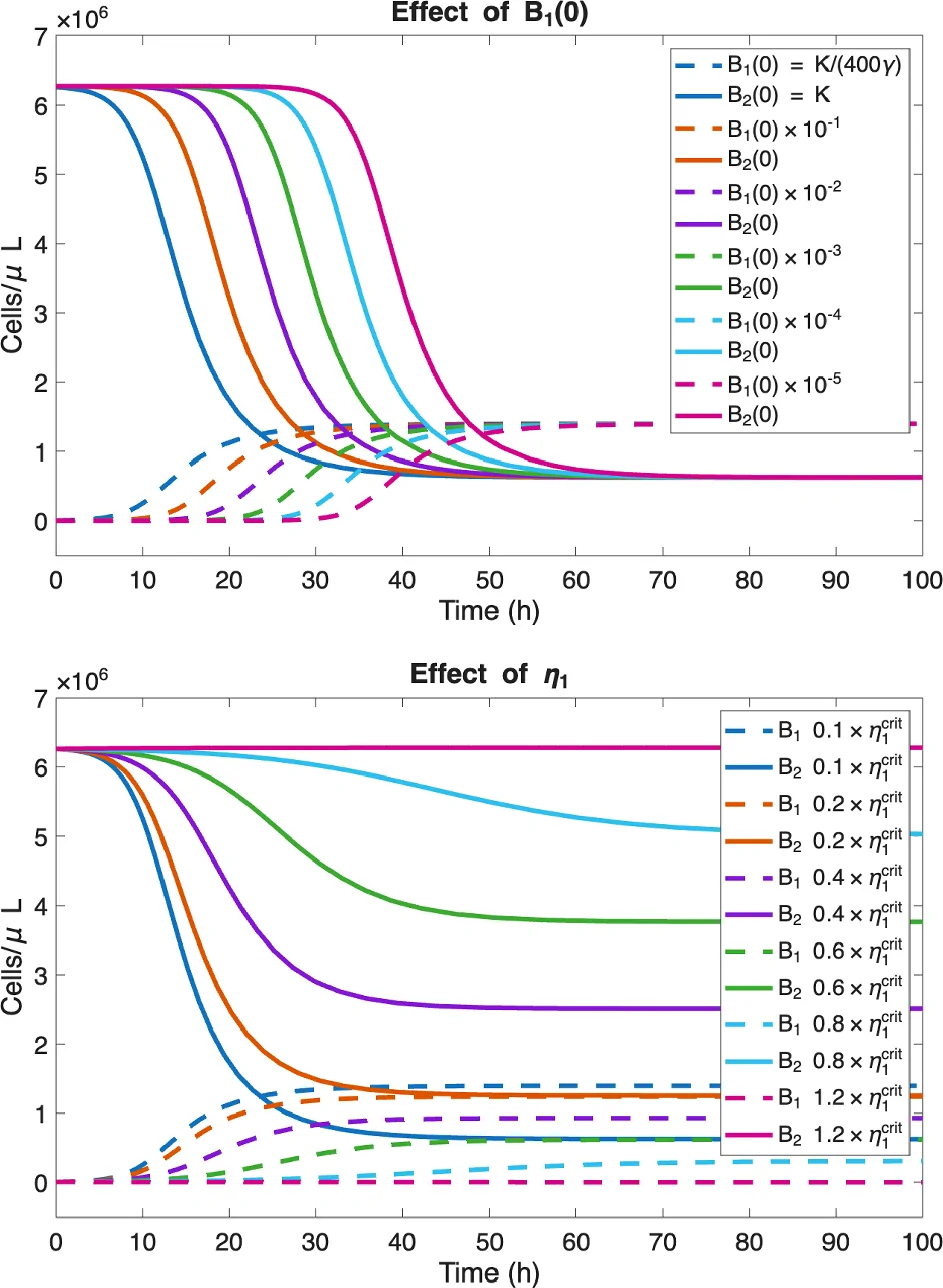
Top: Illustrates the long-term effects of changing the initial amount of plasmid-carrying bacteria, $B_1$, when plasmid-free bacteria, $B_2$, starts at carrying capacity, *K*, and $\eta _1 = 0.1 \eta _1^{crit} = K \beta$. The initial value of $B_1$ increases by factors of 10 from $10^{-5} B_1(0)$ to $B_1(0) = \displaystyle \frac{K}{400 \gamma }$. Each color corresponds to a simulation with a different initial value for $B_1(0).$ Bottom: Illustrates the long-term effects of changing the rate of plasmid loss, $\eta _1$ when $B_1(0) = \displaystyle \frac{K}{400 \gamma }$, with $B_2(0)$ at carrying capacity, *K*. Each color corresponds to a simulation in which the value of $\eta _1 = \textit{coeff}*\eta _1^{crit}$, where $\textit{coeff}=0.1, 0.2, 0.4, 0.6, 0.8, 1.2$. Note that as $\textit{coeff}$ increases it less likely that the two bacterial species, $B_1$ and $B_2$, will coexist (color figure online)

In Figure 9 (top), we explore the effect of introducing varying amounts of plasmid-carrying bacteria, $B_1(0)$, under a constant antibiotic dose, with $B_{2}$ starting at carrying capacity. In this case, $\eta _{1}$, the transition rate from plasmid-carrying bacteria $B_1$ to plasmid-free bacteria $B_2$ is set to $0.1\,\eta _{1}^{\text {crit}}$, where $\eta _{1}^{\text {crit}} = K \beta$. The results indicate that the initial inoculum size affects the system’s transient behavior, but that in all cases, the two populations reach the same coexisting equilibrium after six hours, where the newly-introduced plasmid-carrying bacteria dominates.

In Figure 9 (bottom), the same model is examined while keeping the initial $B_{1}$ concentration constant ($B_{1}(0) = 400/(\gamma K)$) and varying $\eta _{1}$ as a fraction of its critical value, from 0.1 to 1.2. Since the plasmid can confer greater antibiotic resistance, changing the rate of plasmid loss in these simulations allows us to explore the impact of different antibiotic doses on the overall dynamics. The results show that increasing antibiotic concentrations could markedly alter both the conversion rate and the resulting equilibrium between plasmid-carrying and plasmid-free populations. Higher antibiotic doses suppress bacterial growth but also shift the long-term balance between $B_{1}$ and $B_{2}$, highlighting the complex interplay between pharmacological stress and plasmid transfer dynamics.

**Fig. 10:**
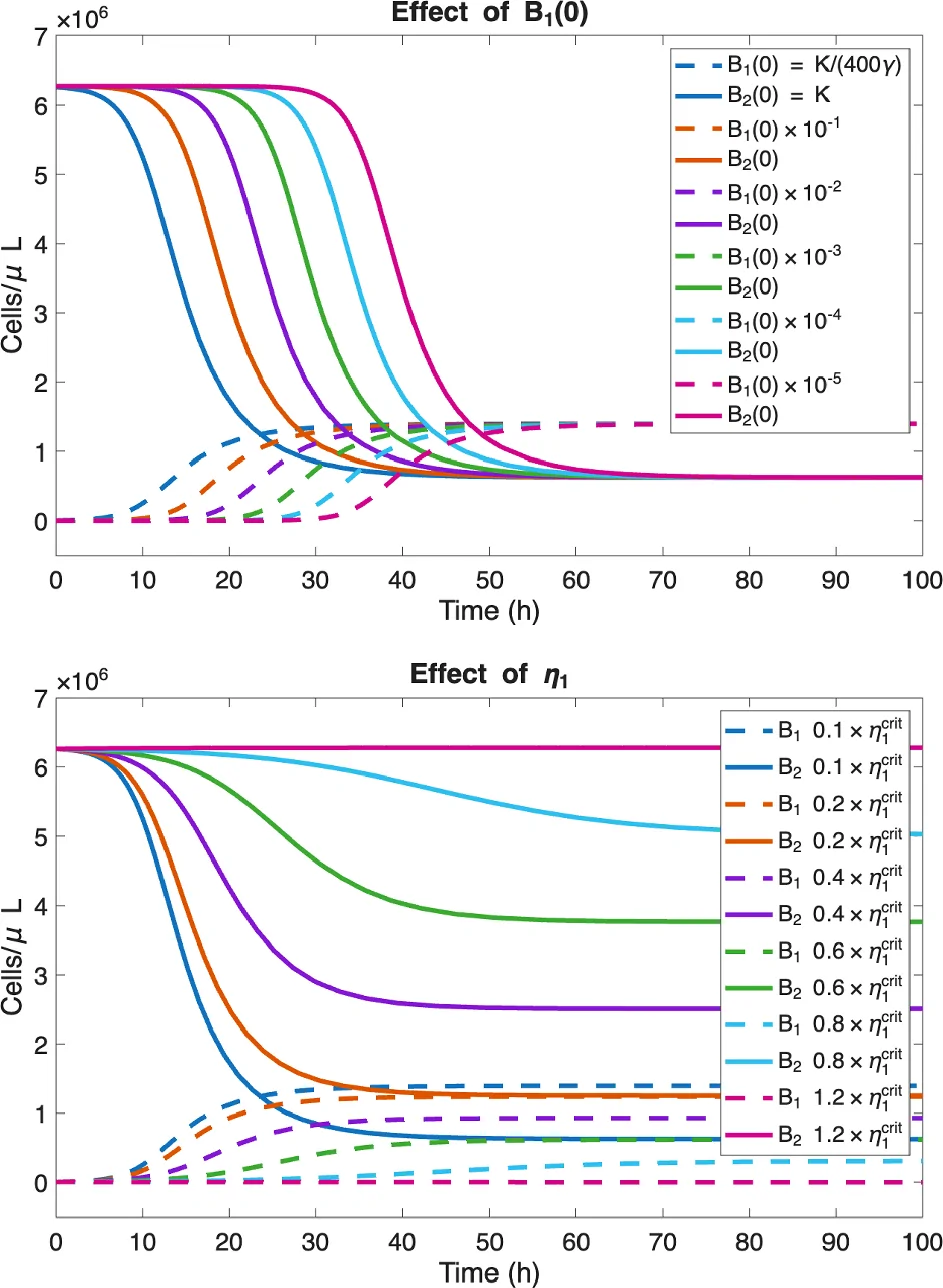
Top: Illustrates the long-term effects of changing the rate of plasmid loss, $\eta _2$, when $B_1(0) = \displaystyle \frac{K}{400 \gamma }$, $B_2(0)=K$, $\eta _1 = 0$, and the conjugation rate, β=0. Values of $\eta _2$ increase from $1.5 \times 10^{-3}$ to 1.5 by factors of 10. Bottom: Illustrates the long-term effects of changing the conjugation rate, β, when $B_1(0) = \displaystyle \frac{K}{400 \gamma }$, $B_2(0)=K$. Values of $\beta = \textit{coeff} *\beta ^0$ increase, as $\textit{coeff}$ increases from 0.25 to 2. Note that as $\textit{coeff}$ decreases the density of $B_2$ increases (color figure online)

Next, Figure 10 looks at how the system changes when $\eta _2$, the conversion rate from $B_2$ to $B_1$ (top of figure), and β, the rate of bacterial conjugation (bottom of figure), are changed. In the instance of $\eta _1=0$, the top figure shows how different $\eta _2$ values affect the system when a fixed amount of plasmid-carrying bacteria $B_1$ is added. Since plasmid-carrying bacteria are assumed to be more resistant to antibiotics, an increase in antibiotics could result in more bacteria taking up plasmid, and more bacteria without plasmid dying. Thus, we can interpret an increase in $\eta _2$ as the result of an increase in antibiotic dose. These simulations show that in all cases the resident, plasmid-free bacteria dies out, and the plasmid-carrying bacteria reach the same equilibrium independent of the value of $\eta _2$. However, increasing $\eta _2$ changes the transient dynamics, allowing the plasmid-carrying bacteria to reach equilibrium in a shorter amount of time.

The bottom picture, on the other hand, shows how changing β, the change from plasmid-free $B_2$ to plasmid-carrying $B_1$ bacteria through conjugation, affects the system. In contrast to the top graph, increasing the rate of conjugation increases the long-term population of plasmid-carrying bacteria $B_1$ and decreases the long-term population of plasmid-free bacteria $B_2$. This suggests that antibiotics, which we assume favors the bacteria with plasmid and increases the conjugation rate, could change the long term-proportions of plasmid-carrying and plasmid-free bacteria. The plot shows concentrations of $B_1$ and $B_2$ over time with $\beta = \textit{coeff} * \beta ^0$. As $\textit{coeff}$ increases from =0.25 to 2, the equilibrium values of the plasmid-carrying bacteria $B_1$ increase, while the equilibrium values of the non-plasmid carrying bacteria $B_2$ decrease. In all simulations, $\beta ^0=7.974 \times 10^{-8}$.

Both results demonstrate that both $\eta _2$ and β are essential in influencing the stability and dynamics of bacterial populations, underscoring the necessity for a thorough understanding of both parameters in the formulation of effective antibiotic therapies.

**Fig. 11:**
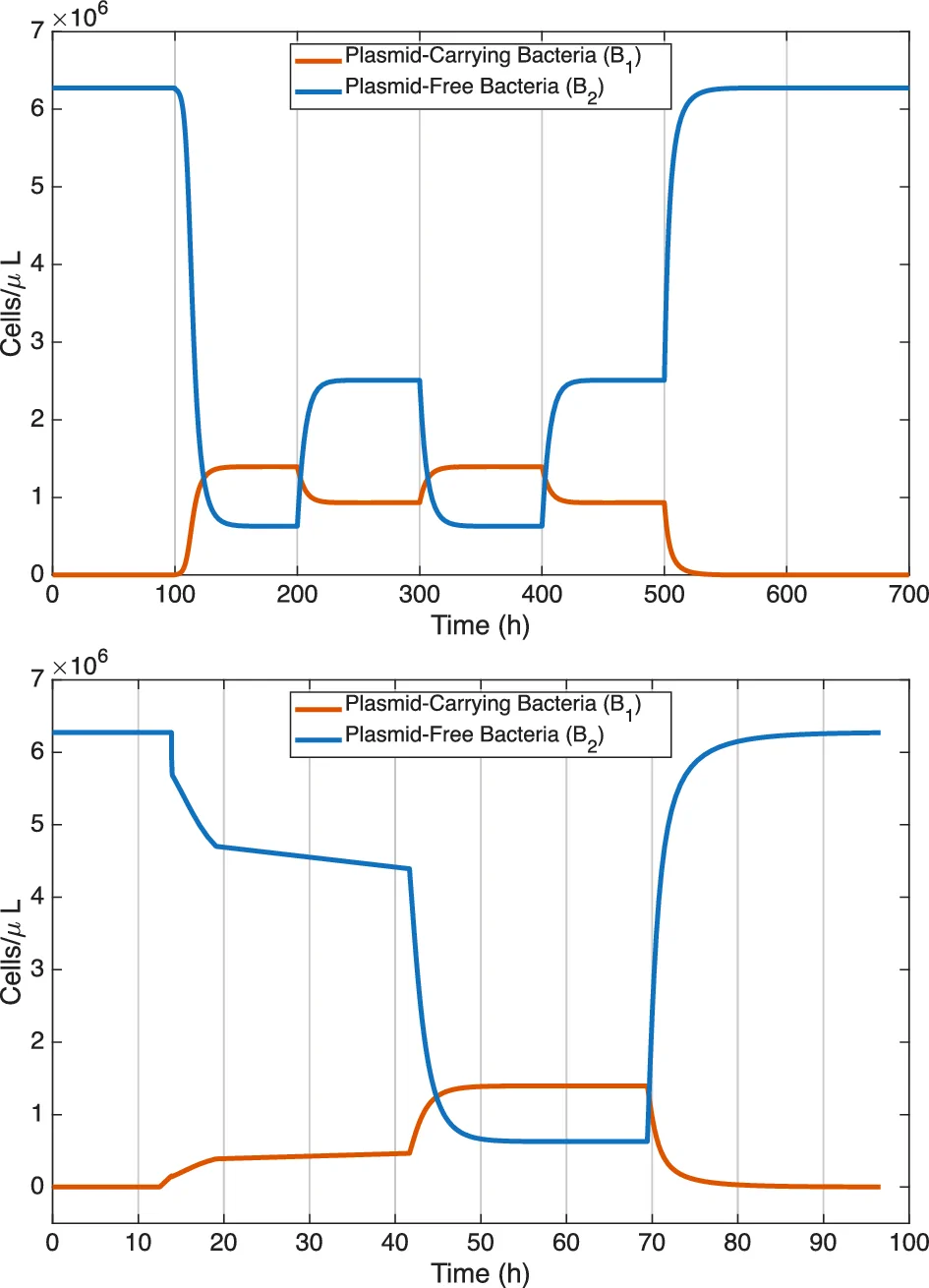
Simulated treatment scenarios illustrating the effect of varying the conversion rate from plasmid-carrying bacteria $B_1$ to plasmid-free bacteria $B_2$, denoted by $\eta _1$. In both panels, simulations start from $B_1(0)=0$ and $B_2(0)=K$ with $\eta _1 > \eta _1^{crit}$. Antibiotic application is modeled through stepwise changes in $\eta _1$, corresponding to different treatment regions. Top: introduction of a small inoculum of plasmid-carrying bacteria combined with alternating high and moderate antibiotic levels applied at 100, 200, 300, 400, and 500 hours. Bottom: dynamics resulting from the introduction of a larger inoculum under a simpler treatment protocol, with antibiotic level changes at 13, 42, and 70 hours (color figure online)

Lastly, Figure 11 shows how changing the rate of conversion ($\eta _1$) from plasmid-carrying bacteria $B_1$ to plasmid-free bacteria $B_2$ affects the results of the experiment in different treatment circumstances. In the top panel, the simulation starts with no plasmid-carrying bacteria while the plasmid-free bacteria is at the carrying capacity of the environment, i.e., initial conditions $B_1 (0)=0, B_2(0)=K$, and a rate of conversion from plasmid-carrying to plasmid-free bacteria, $\eta _1$, above $\eta _1^{crit} = K \beta$ (See Table 3 and Figure 4), i.e., $\eta _1 = 1.2 \eta _1^{crit}$, followed by adding a small amount of plasmid-carrying bacteria ($B_1 =\displaystyle {K}/{400 \gamma }$) at t=100 hours while applying high antibiotic dosage, which reduces $\eta _1$ to $\eta _1 = 0.1 \eta _1^{crit}$, then decreasing the antibiotic at t=200 hours, which raises $\eta _1$ to $\eta _1 = 0.4 \eta _1^{crit}$, followed by increasing the antibiotic again at t=300 hours, which lowers $\eta _1$ back to $\eta _1 = 0.1 \eta _1^{crit}$, and again decreasing the antibiotic at t=400 hours, which restores $\eta _1$ to $\eta _1 = 0.4 \eta _1^{crit}$, before finally removing the antibiotic at t=500 hours, which returns the rate of conversion from plasmid-carrying to plasmid-free bacteria, $\eta _1$, to $\eta _1 = 1.2 \eta _1^{crit}$. In the bottom panel, the simulation starts with no plasmid-carrying bacteria while the plasmid-free bacteria is at the carrying capacity of the environment, i.e., initial conditions $B_1 (0)=0, B_2(0)=K$, and a rate of conversion from plasmid-carrying to plasmid-free bacteria, $\eta _1$, above $\eta _1^{crit}$, i.e., $\eta _1 = 1.1 \eta _1^{crit}$ (Region 1), followed by adding a larger amount of plasmid-carrying bacteria ($B_1 =\displaystyle {K}/{10 \gamma }$) at t=13 hours and applying a small amount of antibiotic, which reduces $\eta _1$ to $\eta _1 = 0.7 \eta _1^{crit}$, then increasing the antibiotic at t=42 hours, which further reduces $\eta _1$ to $\eta _1 = 0.1 \eta _1^{crit}$, and finally removing the antibiotic at t=70 hours, which restores the rate of conversion from plasmid-carrying to plasmid-free bacteria, $\eta _1$, to $\eta _1 = 1.1 \eta _1^{crit}$. This figure shows how antibiotics and changes in the conversion rate work together to affect bacterial populations and shows how antibiotics help control bacterial dynamics.

These figures collectively provide a thorough comprehension of how alterations in treatment strategies–including modulation of conversion rates, conjugation dynamics, and antibiotic administration–affect bacterial population behavior over time. The simulations show how small changes in key biological parameters ($\eta _{1}$, $\eta _{2}$, and β) and external stressors like antibiotics can have a big effect on population stability, coexistence, and the rise of resistance. The results highlight the necessity of integrating both genetic exchange mechanisms and pharmacological effects in the design of antibacterial interventions. They show, in particular, that exposure to antibiotics not only stops bacteria from growing but also changes the balance between plasmid-carrying and plasmid-free strains, which affects the possibility of horizontal gene transfer. This two-pronged effect shows how hard it is for bacteria to adapt to treatment, which shows how important it is to have flexible treatment plans that keep bacteria from growing while also keeping their resistance in check. In general, these findings help us understand how bacteria behave when they are under stress from antibiotics, which gives us a way to improve dosing schedules and lower the long-term risks of resistance spreading.

## Discussion and Conclusions

In this work, we have developed a mathematical model for representing the dynamics of plasmid loss, uptake, and transfer between two bacterial populations (plasmid-carrying bacteria and plasmid-free bacteria). The model is based on batch cultures of *E. coli*, which is pervasive as a bacterial system of choice in biotechnology applications. Here, we find that the population of plasmid-carrying bacteria can reach distinct equilibria based on the rates of bacterial conversion from plasmid-carrying to plasmid-free bacteria. This plasmid loss rate can be controlled by the extent of selection pressure placed on the bacterial system to retain the plasmid. Specifically, a plasmid conferring a benefit for host growth or survival, such as a plasmid bearing an antibiotic resistance gene, would be retained by providing benefit to bacteria when in an environment containing the corresponding antibiotic. Controlling such conditions, such as addition of antibiotic or it’s concentration, can be implemented within *in vitro* (flask batch cultures) and *in vivo* (the microbiome of the gut) bacterial systems. Our model is thus expected to be a useful tool for instance in optimizing cultures for *E. coli* driven formation of products (such as enzyme or protein production by *E. coli* using as expression plasmid system for batch cultures) by guiding selection of an appropriate set of initial seeding and selection conditions as well as predictions for when to add inducers of expression. In addition, by providing a model to predict the extent of plasmid-carrying bacteria within a “one-pot” system, we believe this model may be useful to inform population dynamics related to plasmid retention, loss, and transfer for *in vivo* applications, such as probiotic *E. coli* introduced to the bacterial community of the gut microbiome. In doing so, one could envision using this model as a tool to select an appropriate dosing regimen for probiotics that allows consistent and controlled presence of the plasmid (and its products) within the host while not permanently persisting in the gut. From our results, this could be tailored by ensuring a sufficiently high rate of plasmid loss such that eventually all bacteria are free of the introduced plasmid, as permanent modifications of the gut bacterial function is not desirable. The model results coincide well with experiments utilizing optical density measurement for assessing growth of a plasmid-free *E. coli* batch culture and also a plasmid-carrying *E. coli* batch culture. Specifically, we see lower net growth rate and lower carrying capacity in those cultures of plasmid-carrying *E. coli* which is supported by the experimental results.

While previous studies have examined bacterial growth, the current work helps to predict plasmid dynamics, which we have found to be highly dependent on the benefit *vs.* burden imparted on the bacterial system. Here, the rate of the bacterial loss or gain of a plasmid is deemed consistent within the given experiment allowing predictions of the proportion of bacteria possessing plasmid at any point in time during the culture growth. Future work in this area will explore if these rates may change over time but requires additional experiments which may require instrumentation with finer resolution in quantifying bacterial populations with and without plasmid as a function of time. With the model presented here, we can provide valuable information about the heterogeneity within a batch cell culture system for predicting the abundance of plasmid-carrying populations. The practical implication of this work may help in the design of stable and productive culture systems for generating biological products. Similarly, this work may provide a starting point for improving our ability to predict and control bacterial growth processes in which plasmid persistence or loss are critical aspects of performance of the bacterial system.

## Data Availability

Data sets generated during the current study are available from the corresponding author on reasonable request.

## Appendix A. Stability Analysis of the Equilibria of the Models

We present a detailed existence and stability analysis of the equilibria of System (3.1):

$$\begin{aligned} \begin{array}{rcl} \displaystyle \frac{dB_1}{dt}& =& \displaystyle {\alpha _1B_1\left( 1-\frac{\gamma B_1+B_2}{K}\right) } -{\eta _1 B_1} +{\eta _2 B_2} +{\beta B_1B_2},\\ \\ \displaystyle \frac{dB_2}{dt}& =& \displaystyle {\alpha _2B_2\left( 1-\frac{\gamma B_1+B_2}{K}\right) } +{\eta _1 B_1} -{\eta _2 B_2} -{\beta B_1B_2}. \end{array} \end{aligned}$$

The analysis results depend on the types of bacterial switching present in the system, which are based on the values of the model parameters $\eta _1, \eta _2$ and β. Here, we present the existence and stability analysis of System (3.1). We have four types of equilibria that are biologically relevant:

- Type I: No bacteria are present.
- Type II: Only $B_1$, the plasmid-carrying bacteria, exists.
- Type III: Only $B_2$, the plasmid-free bacteria, exists.
- Type IV: Both bacteria exist in non-zero quantities.

To solve for our equilibria, we start by setting the right hand sides of (3.1) equal to zero. We found five biologically feasible equilibria: $E^0$ to $E^4$ with details on their stability below. Note that the values for $B_1$ and $B_2$ that appear in $E^i$, where i=0,1,2,3,4, represent population sizes. Hence, these values must all be non-negative. We did not include any equilibria found from solving the equations above that that clearly displayed negative populations of bacteria as they are not biologically feasible.

### Appendix A.1. Type I

Our first equilibrium is $E^0 =\left( 0,0\right)$. Hence, $E^0$ is a biologically feasible equilibrium of Type I, where both bacteria populations are zero. The proof of the instability of this equilibrium is below.

#### Theorem Appendix A.1

The boundary equilibrium $E^0$ is unstable.

#### Proof

Assume $\beta ,\eta _2>0$. The Jacobian evaluated at $E^0$ is

$$\begin{aligned} J\left( E^0\right)&=\begin{bmatrix} \alpha _1-\eta _1 & \eta _2\\ \eta _1& \alpha _2-\eta _2 \end{bmatrix}. \end{aligned}$$

Thus,

$$\displaystyle {det\left( {J\left( E^0\right) }\right) }=\displaystyle {(\alpha _1-\eta _1)(\alpha _2-\eta _2)-\eta _1\eta _2}=\alpha _1(\alpha _2-\eta _2)-\alpha _2\eta _1$$

and

$$tr\left( {J\left( E^0\right) }\right) =\displaystyle {(\alpha _1-\eta _1)+(\alpha _2-\eta _2)}.$$

By the Routh-Hurwitz stability criterion (Otto and Day 2011), $E^0$ is stable when $det\left( {J\left( E^0\right) }\right) >0$ and $tr\left( {J\left( E^0\right) }\right) <0$. The above conditions are never satisfied simultaneously. Suppose

$$ det\left( {J\left( E^0\right) }\right) =(\alpha _1-\eta _1)(\alpha _2-\eta _2)-\eta _1\eta _2 =\alpha _1(\alpha _2-\eta _2)-\alpha _2\eta _1 >0 $$

and

$$ tr\left( {J\left( E^0\right) }\right) =(\alpha _1-\eta _1)+(\alpha _2-\eta _2) <0.$$

This implies, $(\alpha _1-\eta _1)(\alpha _2-\eta _2)>\eta _1\eta _2>0$ and $\alpha _1(\alpha _2-\eta _2)>\alpha _2\eta _1 >0$. Thus, $(\alpha _1-\eta _1)>0 \text { and } (\alpha _2-\eta _2) >0$. Lastly, $tr\left( {J\left( E^0\right) }\right) =(\alpha _1-\eta _1)+(\alpha _2-\eta _2) >0$. A contradiction has been reached.

If $\eta _2>0$ and β=0, an identical proof would follow. If $\eta _2=0$ and β>0, a similar proof would follow. In conclusion, the Routh-Hurwitz stability criterion is never satisfied, and $E^0$ is always unstable. □

### Appendix A.2. Type II

Our second equilibrium occurs when $\eta _1 = 0$ and $B_2 = 0$. In that case, $\displaystyle E^1 = \left( \frac{K}{\gamma }, 0\right)$. Note that K>0, so we must have γ>0 for this equilibrium to be biologically feasible. In this case, only the plasmid-carrying bacteria are present. The proof that $E^1$ is asymptotically stable when $\eta _1 = 0$ is below.

#### Theorem Appendix A.2

The boundary equilibrium $E^1 = \left( \displaystyle \frac{K}{\gamma }, 0\right)$ is locally asymptotically stable when $\eta _1 = 0$.

#### Proof

Assume that $\eta _2$ or β is non-zero in order for our system to not collapse into a well studied system. Evaluating the Jacobian at $E^1$, we see

$$ J\left( E^1\right) =\begin{bmatrix} -\alpha _1 & -\dfrac{\alpha _1}{\gamma } + \eta _2 + \dfrac{\beta K}{\gamma }\\ 0 & -\eta _2 - \dfrac{\beta K}{\gamma } \end{bmatrix}. $$

By the Routh-Hurwitz stability criterion (Otto and Day 2011), $E^1$ is stable when $det\left( {J\left( E^1\right) }\right) >0$ and $tr\left( {J\left( E^1\right) }\right) <0$. Since our parameters $\alpha _1, K, \gamma >0$,

$$ det\left( {J\left( E^1\right) }\right) = \alpha _1\left( \eta _2 + \frac{\beta K }{\gamma }\right) > 0. $$

Our trace

$$ tr\left( {J\left( E^1\right) }\right) = -\alpha _1 -\eta _2 -\frac{\beta K}{\gamma }< 0 $$

since our parameters $\alpha _1, K, \gamma >0$ and $\beta \geqslant 0$. Thus, $E^1$ is an asymptotically stable equilibrium. □

### Appendix A.3. Type III

Our third equilibrium occurs when $\eta _2 = 0$ and $B_1 = 0$. In that case, $E^2 = (0, K)$. Note that K>0 and, thus, only the plasmid-free bacteria exist. $E^2$ is asymptotically stable when $\beta K - \eta _1 <0$, otherwise it unstable, as shown below.

#### Theorem Appendix A.3

The boundary equilibrium $E^2 = (0, K)$ exists when $\eta _2 = 0$ and is locally asymptotically stable if and only if $\beta K - \eta _1 <0$.

#### Proof

Assume $\eta _2=0$ and $\beta K - \eta _1 <0$. The Jacobian evaluated at $E^2$ is

$$ J(E^2)=\begin{bmatrix} \beta K-\eta _1& 0\\ \eta _1-\gamma \alpha _2& -\alpha _2 \end{bmatrix}. $$

By the Routh-Hurwitz stability criterion (Otto and Day 2011), $E^2$ is stable when $det\left( {J\left( E^2\right) }\right) >0$ and $tr\left( {J\left( E^2\right) }\right) <0$. Since $\displaystyle {\beta K-\eta _1<0}$ and we are assuming that $\alpha _2 > 0$, it follows directly that:

$$\displaystyle {det\left( {J\left( E^2\right) }\right) }=\displaystyle {-(\beta K-\eta _1)\alpha _2}>0$$

and

$$tr\left( {J\left( E^2\right) }\right) =\displaystyle {(\beta K-\eta _1)-\alpha _2}<0.$$

Thus, $E^2$ is locally asymptotically stable if and only if $\eta _2=0$ and $\displaystyle {\beta K-\eta _1<0}$. □

### Appendix A.4. Type IV

The following equilibria are of Type IV; that is, both bacteria exist for some non-zero quantity under specific circumstances based on the parameters chosen. Again, we are searching for biologically relevant equilibria.

#### Appendix A.4.1. Equilibrium $E^3$

Our fourth equilibrium is $E^3 = \left( B_1^1,B_2^1\right)$ where

$$\begin{aligned} B_1^1=\frac{K\beta -(\eta _1+\gamma \eta _2)+\sqrt{4K\gamma \eta _2\beta +(\eta _1+\gamma \eta _2-K\beta )^2}}{2\beta \gamma }, \\ B_2^1=\frac{K\beta +(\eta _1+\gamma \eta _2)-\sqrt{4K\gamma \eta _2\beta +(\eta _1+\gamma \eta _2-K\beta )^2}}{2\beta }, \end{aligned}$$

Observe that when β=0, the denominators of $B_1^1$ and $B_2^1$, $2\beta \gamma$ and 2β, are zero. Thus, if β=0, our fourth equilibrium is undefined and not an equilibrium of System (3.1). Hence, we must have β>0. Now, we will show that this equilibrium is biologically feasible.

Assume $\eta _2>0$. Notice that $B_1^1$’s denominator, $2\beta \gamma$, is positive. Therefore, $B_1^1$ is non-negative if and only if $B_1^1$’s numerator is non-negative. Note that the following inequalities hold.

$$\begin{aligned} (K\beta -\eta _1-\gamma \eta _2)^2&<(K\beta -\eta _1-\gamma \eta _2)^2+4K\beta \gamma \eta _2\\ -(K\beta -\eta _1-\gamma \eta _2)\leqslant |K\beta -\eta _1-\gamma \eta _2|&<\sqrt{(K\beta -\eta _1-\gamma \eta _2)^2+4K\beta \gamma \eta _2}\\ 0<(K\beta -\eta _1-\gamma \eta _2)&+\sqrt{(K\beta -\eta _1-\gamma \eta _2)^2+4K\beta \gamma \eta _2} \end{aligned}$$

Thus, $B_1^1$’s numerator and, consequently, $B_1^1$ are non-negative.

Notice that $B_2^1$’s denominator, 2β, is positive. Therefore, $B_2^1$ is non-negative if and only if $B_2^1$’s numerator is non-negative. Notice

$$\begin{aligned} (K\beta +\eta _1+\gamma \eta _2)^2&>(K\beta +\eta _1+\gamma \eta _2)^2-4K\beta \eta _1\\ K\beta +\eta _1+\gamma \eta _2=|K\beta +\eta _1+\gamma \eta _2|&>\sqrt{(K\beta +\eta _1+\gamma \eta _2)^2-4K\beta \eta _1}\\ K\beta +\eta _1+\gamma \eta _2&-\sqrt{(K\beta +\eta _1+\gamma \eta _2)^2-4K\beta \eta _1}>0 \end{aligned}$$

Since

$$ (K\beta +\eta _1+\gamma \eta _2)^2-4K\beta \eta _1 = (\eta _1+\gamma \eta _2-K\beta )^2 + 4K\gamma \eta _2\beta , $$

$K\beta +(\eta _1+\gamma \eta _2)-\sqrt{4K\gamma \eta _2\beta +(\eta _1+\gamma \eta _2-K\beta )^2}>0$. Thus, $B_2^1$’s numerator and, consequently, $B_2^1$ are non-negative. Thus, when $\beta , \eta _2>0$, $E^3$ exists and is biologically feasible.

When $\eta _2 = 0$, our equilibrium is of the form:

$$\begin{aligned} \displaystyle {B_1^1=\frac{K\beta -\eta _1+\sqrt{(\eta _1-K\beta )^2}}{2\beta \gamma }}, \\ \displaystyle {B_2^1=\frac{K\beta +\eta _1-\sqrt{(\eta _1-K\beta )^2}}{2\beta }}, \end{aligned}$$

which reduces to either

$$ \left( B_1^1,B_2^1\right) =\displaystyle \left( \frac{\beta K-\eta _1}{\gamma \beta },\frac{\eta _1}{\beta } \right) $$

or

$$ \left( B_1^1,B_2^1\right) = (0, K). $$

In both cases, these equilibria are biologically feasible. Thus, $E^3$ is biologically feasible when β>0. The proof of stability of this equilibrium is shown below. Note that the proof is divided into three cases: 1) $\eta _2 >0$, 2) $\eta _2 = 0$ and $\beta K - \eta _1>0$, and 3) $\eta _2 = 0$ and $\beta K - \eta _1<0$.

**Case 1** $$\eta _2>0$$

##### Theorem Appendix A.4

The interior equilibrium $E^3$ is locally asymptotically stable when $\beta ,\eta _2>0$.

##### Proof

Assume $\beta ,\eta _2>0$. The Jacobian evaluated at $E^3$ is

$$ J\left( E^3\right) =\begin{bmatrix}\displaystyle {\beta B_2^1-\frac{\alpha _1}{K}B_2^1-2\frac{\alpha _1\gamma }{K}B_1^1+\alpha _1-\eta _1}& \displaystyle {\left( \beta -\frac{\alpha _1}{K}\right) B_1^1+\eta _2}\\ \\ \displaystyle {\left( -\beta -\frac{\alpha _2\gamma }{K}\right) B_2^1+\eta _1}& \displaystyle {-\beta B_1^1-\frac{\alpha _2\gamma }{K}B_1^1-2\frac{\alpha _2}{K}B_2^1+\alpha _2-\eta _2}\end{bmatrix}. $$

By the Routh-Hurwitz stability criterion (Otto and Day 2011), $E^3$ is stable when $det\left( {J\left( E^3\right) }\right) >0$ and $tr\left( {J\left( E^3\right) }\right) <0$. Clearly

$$\begin{aligned} det\left( J\left( E^3\right) \right) = \dfrac{\sqrt{(\eta _1+\gamma \eta _2-K\beta )^2+4K\beta \gamma \eta _2}}{K}\left( \alpha _1 B_1^1+\alpha _2B_2^1\right) >0 \end{aligned}$$

and

$$\begin{aligned} tr\left( J\left( E^3\right) \right) =&\displaystyle {\frac{1}{2}X-\beta B_1^1-\frac{\alpha _1\gamma }{K}B_1^1-\frac{\alpha _2}{K}B_2^1}-\eta _2<0 \end{aligned}$$

since

$$\begin{aligned} X&=\displaystyle {K\beta -\eta _1+\gamma \eta _2-\sqrt{(K\beta -\eta _1+\gamma \eta _2)^2+4\gamma \eta _1\eta _2}}<0, \end{aligned}$$

as shown below:

$$\begin{aligned} (K\beta -\eta _1+\gamma \eta _2)^2&<(K\beta -\eta _1+\gamma \eta _2)^2+4\gamma \eta _1\eta _2\\ K\beta -\eta _1+\gamma \eta _2\leqslant |K\beta -\eta _1+\gamma \eta _2|&<\sqrt{(K\beta -\eta _1+\gamma \eta _2)^2+4\gamma \eta _1\eta _2}\\ X=K\beta -\eta _1+\gamma \eta _2&-\sqrt{(K\beta -\eta _1+\gamma \eta _2)^2+4\gamma \eta _1\eta _2}<0. \end{aligned}$$

Thus, $E^3$ is locally asymptotically stable when $\beta ,\eta _2>0$. □

**Case 2** $$\beta>0,\,\eta _2=0,\,\beta K-\eta _1>0$$

##### Theorem Appendix A.5

The interior equilibrium $E^3 =\left( B_1^1,B_2^1\right) =\displaystyle \left( \frac{\beta K-\eta _1}{\gamma \beta },\frac{\eta _1}{\beta } \right)$ where $\beta >0, \eta _2=0$, and $\beta K-\eta _1>0$ is stable whenever it exists.

##### Proof

Assume $\eta _2=0$ and $\beta K-\eta _1>0$. The Jacobian evaluated at $E^3$ is

$$ J(E^3)=\begin{bmatrix} \alpha _1\left( \dfrac{\eta _1}{K\beta }-1\right) & \dfrac{(\beta K-\alpha _1)(\beta K-\eta _1)}{\beta \gamma K}\\ -\dfrac{\alpha _2\eta _1\gamma }{\beta K} & \eta _1\left( \dfrac{1}{\gamma }-\dfrac{\alpha _2}{\beta K}\right) -\dfrac{\beta K}{\gamma } \end{bmatrix}. $$

By the Routh-Hurwitz stability criterion (Otto and Day 2011), $E^3$ is stable when $det\left( {J\left( E^3\right) }\right) >0$ and $tr\left( {J\left( E^3\right) }\right) <0$. Since $\displaystyle {0<\beta K-\eta _1}$, clearly

$$ \det \left( {J\left( E^3\right) }\right) = \frac{(\beta K-\eta _1)[\alpha _1(\beta K-\eta _1)+\alpha _2\eta _1\gamma ]}{\beta \gamma K}>0 $$

and

$$ tr\left( {J\left( E^3\right) }\right) = \frac{-\alpha _2\eta _1\gamma -\alpha _1\gamma (\beta K-\eta _1)-\beta K(\beta K-\eta _1)}{\beta \gamma K}<0. $$

Thus, $E^3$ is locally asymptotically stable if $\eta _2=0$ and $\displaystyle {0<\beta K-\eta _1}$. □

**Case 3** $$\beta >0, \, \eta _2=0, \,\beta K-\eta _1<0$$

In this case, $E^3$ and $E^2$ coincide, and the equilibrium is locally asymptotically stable. See the proof in Appendix A.3.

In summary, $E^3$ is biologically feasible and exists when β>0. We have a Type IV equilibrium when β>0 and $\eta _2>0$ that is asymptotically stable. When β>0, $\eta _2 = 0$, and $\beta K-\eta _1>0$, we have another Type IV, stable equilibrium.

Note two special cases of this equilibrium: 1) $\eta _1 = 0$ and 2) $\eta _2 = 0$ and $\beta K - \eta <0$. When $\eta _1 = 0$, the stable, Type II (only plasmid-carrying bacteria exist) equilibrium appears $(\frac{K}{\gamma },0)$. When $\eta _2 = 0$ and $\beta K - \eta <0$, the stable Type III (only plasmid-free bacteria exist) equilibrium appears (0, *K*).

#### Appendix A.4.2. Equilibrium $E^4$

Our fifth equilibrium $E^4$ occurs when β=0 where

$$ E^4 =\left( B_1^3,B_2^3\right) =\left( \frac{\eta _2K}{\eta _1+\gamma \eta _2},\frac{\eta _1K}{\eta _1+\gamma \eta _2}\right) $$

Note that $\eta _1+\gamma \eta _2 >0$ for the equilibrium to be biologically feasible. If $\eta _1 = 0$, we reduce down to the equilibrium $\left( \frac{K}{\gamma }, 0\right)$, in Appendix A.2. If $\eta _2 = 0$, we reduce down to the equilibrium $\left( 0, K\right)$, in Appendix A.3.

Assume $\eta _1, \eta _2>0$. Then, $\displaystyle {B_1^3=\frac{K\eta _2}{\eta _1+\eta _2\gamma }}$ and $\displaystyle {B_2^3=\frac{K\eta _1}{\eta _1+\eta _2\gamma }}$ are non-negative. Therefore, $E^4$ with $\eta _1, \eta _2>0$ is an asymptotically stable, biologically feasible equilibrium of Type IV. The proof of stability is below.

##### Theorem Appendix A.6

The interior equilibrium $E^4$ is locally asymptotically stable when $\eta _1, \eta _2>0$.

##### Proof

The Jacobian evaluated at $E^4$ is

$$ J(E^4)=\begin{bmatrix} \displaystyle {-\frac{\eta _1^2+\alpha _1\gamma \eta _2+\gamma \eta _1\eta _2}{\eta _1+\gamma \eta _2}} & \displaystyle {\eta _2-\frac{\alpha _1\eta _2}{\eta _1+\gamma \eta _2}}\\ \displaystyle {\eta _1-\frac{\alpha _2\gamma \eta _1}{\eta _1+\gamma \eta _2}}& \displaystyle {-\eta _2-\frac{\alpha _2\eta _1}{\eta _1+\gamma \eta _2}} \end{bmatrix}. $$

By the Routh-Hurwitz stability criterion (Otto and Day 2011), $E^3$ is stable when $det\left( {J\left( E^4\right) }\right) >0$ and $tr\left( {J\left( E^4\right) }\right) <0$. Clearly,

$$ det(J(E^4))=\alpha _2\eta _1+\alpha _1\eta _2>0, $$

and

$$ tr(J(E^4))=-\frac{\alpha _2\eta _1+\eta _1^2+(1+\gamma )\eta _1\eta _2+\gamma \eta _2(\alpha _1+\eta _2)}{\eta _1+\gamma \eta _2}<0. $$

Thus, $E^4$ is locally asymptotically stable when $\eta _1, \eta _2>0$. □

### Appendix A.5. Summary of equilibria

A summary of the equilibria in table form is below in Table 7 and Table 8. Table 7 displays a summary of the for equilibria under different parameter regimes, along with their stability. Table 8 illustrates the types of equilibria under each parameter regime we considered.

**Table 7:** Existence and local stability results for the equilibrium points of System (3.1). A red cell represents an unstable equilibrium, and a green cell represents a stable equilibrium. Note that an equilibrium may not be biologically feasible under certain parameter regimes or not exist and is labeled as “does not exist"

**Table 8:** Interpretation of the equilibrium points of System (3.1). Maroon cells represent Type I equilibria. Yellow cells represent Type II, and blue cells Type III equilibria. Bright green cells represent Type IV equilibria

### Appendix A.6. Proof of global stability of $E^1$

The following lemma shows that System (3.1) is dissipative, and if $B_i(0)\geqslant 0,$ then $B_i(t)\geqslant 0$ for all t⩾0 and i=1,2.

#### Lemma Appendix A.1

The region $\Omega =\left\{ (B_1,B_2): B_1\geqslant 0,B_2\geqslant 0, B_1+B_2\leqslant \frac{K}{\gamma }\right\}$ is positively invariant with respect to System (3.1). Moreover, System (3.1) is dissipative.

#### Proof

Since $E^0=(0,0)$ is an equilibrium of System (3.1), then if $B_1(0)\geqslant 0$ and $B_2(0)\geqslant 0,$ then $B_1(t)\geqslant 0$ and $B_2(t)\geqslant 0.$ Let $\Sigma =B_1+B_2.$ Notice, $\alpha _1<\alpha _2$ and γ>1. Thus,

$$\begin{aligned} \Sigma '=B'_1+B'_2=\Big (\alpha _1B_1+\alpha _2B_2\Big )\Big (1-\frac{\gamma B_1+ B_2}{K}\Big )&<\\ \Big (\alpha _1B_1+\alpha _2B_2\Big )\Big (1-\frac{ B_1+ B_2}{K}\Big )&<\\ \alpha _2(B_1+B_2)\Big (1-\frac{B_1+B_2}{K}\Big )=\alpha _2\Sigma \Big (1-\frac{\Sigma }{K}\Big ). \end{aligned}$$

Therefore, the region Ω is positively invariant, and System (3.1) is dissipative on Ω. □

#### Theorem Appendix A.7

The equilibrium $E^1$ is globally asymptotically stable on Ω.

#### Proof

From Lemma (Appendix A.1) the solution $(B_1(t),B_2(t))$ of System (3.1) is positive and bounded. Since $E^1$ is locally asymptotically stable, then by Poincaré-Bendixson theorem it suffices to show System (3.1) does not have periodic solutions. To prove this we use the Dulac criterion. We define

$$ H(B_1,B_2)=\frac{1}{B_1B_2},\;\;B_1>0,\;\;B_2>0. $$

Let

$$\begin{aligned} f_1(B_1,B_2)&=\alpha _1B_1\Big (1-\frac{\gamma B_1+B_2}{K}\Big )-\eta _1 B_1 +\eta _2 B_2+\beta B_1B_2,\\ f_2(B_1,B_2)&=\alpha _2B_2\Big (1-\frac{\gamma B_1+B_2}{K}\Big )+\eta _1 B_1 -\eta _2 B_2-\beta B_1B_2. \end{aligned}$$

From (3.1), a straightforward computation yields:

$$ \frac{\partial (f_1H)}{\partial B_1}+\frac{\partial (f_2H)}{\partial B_2}=-\frac{\alpha _2B_1B^2_2+B^2_1(\alpha _1\gamma B_2+\eta _1K)+\eta _2KB^2_2}{KB^2_1B^2_2}<0. $$

Hence, there are no nontrivial periodic solutions, and the proof is completed. □

## Code Availability

Code to replicate the analysis supporting the findings of this manuscript are available from the corresponding author on reasonable request.
